# A microalgal‐based preparation with synergistic cellulolytic and detoxifying action towards chemical‐treated lignocellulose

**DOI:** 10.1111/pbi.13447

**Published:** 2020-09-02

**Authors:** Manuel Benedetti, Simone Barera, Paolo Longoni, Zeno Guardini, Natalia Herrero Garcia, David Bolzonella, Damar Lopez‐Arredondo, Luis Herrera‐Estrella, Michel Goldschmidt‐Clermont, Roberto Bassi, Luca Dall’Osto

**Affiliations:** ^1^ Dipartimento di Biotecnologie Università di Verona Verona Italy; ^2^ Faculty of Science Institute of Biology University of Neuchâtel Neuchâtel Switzerland; ^3^ StelaGenomics Mexico S de RL de CV Irapuato, Guanajuato Mexico; ^4^ Laboratorio Nacional de Genómica para la Biodiversidad Centro de Investigación y de Estudios Avanzados del Instituto Politécnico Nacional Irapuato, Guanajuato Mexico; ^5^ Institute of Genomics for Crop Abiotic Stress Tolerance Texas Tech University Lubbock TX USA; ^6^ Department of Botany and Plant Biology University of Geneva Geneva Switzerland; ^7^ Present address: Dipartimento MESVA Università dell'Aquila Coppito AQ Italy

**Keywords:** biofuel, biogas, cell wall degrading enzymes, Chlamydomonas, Chlorella, glycoside hydrolases, phosphite, plant cell wall, transplastomic microalgae

## Abstract

High‐temperature bioconversion of lignocellulose into fermentable sugars has drawn attention for efficient production of renewable chemicals and biofuels, because competing microbial activities are inhibited at elevated temperatures and thermostable cell wall degrading enzymes are superior to mesophilic enzymes. Here, we report on the development of a platform to produce four different thermostable cell wall degrading enzymes in the chloroplast of *Chlamydomonas reinhardtii*. The enzyme blend was composed of the cellobiohydrolase CBM3GH5 from *C*.* saccharolyticus*, the β‐glucosidase celB from *P*.* furiosus*, the endoglucanase B and the endoxylanase XynA from *T*.* neapolitana*. In addition, transplastomic microalgae were engineered for the expression of phosphite dehydrogenase D from *Pseudomonas stutzeri,* allowing for growth in non‐axenic media by selective phosphite nutrition. The cellulolytic blend composed of the glycoside hydrolase (GH) domain GH12/GH5/GH1 allowed the conversion of alkaline‐treated lignocellulose into glucose with efficiencies ranging from 14% to 17% upon 48h of reaction and an enzyme loading of 0.05% (w/w). Hydrolysates from treated cellulosic materials with extracts of transgenic microalgae boosted both the biogas production by methanogenic bacteria and the mixotrophic growth of the oleaginous microalga *Chlorella vulgaris*. Notably, microalgal treatment suppressed the detrimental effect of inhibitory by‐products released from the alkaline treatment of biomass, thus allowing for efficient assimilation of lignocellulose‐derived sugars by *C*.* vulgaris* under mixotrophic growth.

## Introduction

Lignocellulose, the most abundant organic carbon source on Earth, has great potential for conversion into renewable fuels. However, the lack of methods for its efficient hydrolysis to easily fermentable sugars limits such potential (Saini *et al*., 2015; Sanderson, 2011). Different methods including physical, chemical and biological pretreatments have been employed for enhancing lignocellulose degradation (Badiei *et al*., 2014; Harmsen *et al*., 2010; Kumar and Sharma, 2017). Physical pretreatments include thermal, microwave and ultrasounds treatments (Battista *et al*., 2015; Ren *et al*., 2018; Savoo and Mudhoo 2018), promoting substrate disaggregation and breaking of large molecules into smaller oligomers for digestion by microorganisms. Chemical pretreatments are harmful for the environment and negatively impact the rationale of using lignocellulose to produce cleaner forms of fuels. Furthermore, such treatments generate by‐products that inhibit the microbial fermentation of lignocellulose‐derived sugars, thus reducing the conversion yield into biofuel‐related compounds (Jönsson and Martín 2016). Biological treatments include the use of microbial cell wall degrading enzymes (CWDEs), which are currently obtained by culturing mesophilic fungi and bacteria with lignocellulolytic activities (Sánchez 2009). In general, such organisms secrete a wide array of CWDEs in low amounts, as they are strictly required for their own livelihood.

Competitive industrial production should combine high productivity (understood as high quantity of enzymes expressed per day) at a low production cost. A strain that fits these traits would be a valuable candidate for large‐scale expression of CWDEs. From this perspective, transgenic microalgae have the potential of becoming biofactories on an industrial scale due to their relatively fast growth on low‐cost media, including wastewaters and agro‐industrial waste (Benedetti *et al*. 2018a; Brasil *et al.,* 2017). However, genetic engineering of microalgae still lags behind other microorganisms and it presents several constraints, including poor technological development of microalgae as a heterologous expression system, which strongly limits their application as bioreactors. Among the factors that negatively impact nuclear expression of proteins in microalgae, gene silencing can play a prominent role (Schroda 2006). However, the nuclear expression of recombinant proteins was further improved in last years, allowing to reach higher yields than those previously reported (Lauersen *et al*. 2013; Ramos‐Martinez *et al*. 2017), including the expression of fungal xylanases (Rasala *et al*. 2012). The chloroplast of *C*.* reinhardtii* was also used as a biofactory for the production of thermophilic endoglucanases (Faè *et al*. 2017; Richter *et al*. 2018). Chloroplast expression has a number of advantages for recombinant protein production compared with nuclear transformation, including precise transgene integration, absence of gene silencing and high transgene copy number (Mayfield *et al*. 2007). Moreover, since CWDEs of bacterial origin do not require post‐translational modification for proper functioning, the algal transplastomic system appears ideal for their expression.

Arrays of CWDEs need to be produced by cellulolytic fungi and bacteria to achieve efficient degradation of cellulose (Horn *et al*. 2012). CWDEs constitute a highly heterogeneous family, divided into many subcategories and classes (Choi *et al*. 2013; Kubicek *et al*. 2014). Degradation of cellulose by mesophilic organisms involves glycosyl‐hydrolases and oxidoreductases (Dimarogona *et al.,* 2012), which synergistically act to efficiently degrade amorphous and crystalline regions of cellulose, respectively. Glycosyl‐hydrolases include endo‐, exo‐glucanases and β‐glucosidases. The endoglucanases cleave cellulose through a multi‐chain attack mode, generating fragments with different degrees of polymerization. Concomitantly, the exo‐acting glycosyl‐hydrolases, such as cellobiohydrolases, depolymerize such fragments into cellobiose units, which, in turn, are converted into glucose by the β‐glucosidases (Singhania *et al.,* 2013). The crystalline region of cellulose is the main target of cellulolytic oxidases. Among them, lytic polysaccharide mono‐oxygenase (LPMO) disrupts cellulose fibres by oxidative cleavages, thus enhancing the action of cellulolytic hydrolases (Laurent *et al*. 2019; Villares *et al*. 2017).

When compared to the use of their mesophilic counterparts, hyperthermophilic CWDEs (HCWDEs) have several advantages for degradation of plant biomass, which are mainly dependent on the high temperature at which HCWDEs exert the activity (Anitori 2012; Peng *et al*. 2015). High temperature promotes partial detachment of lignin from the hemicellulose–cellulose assembly favouring the hydrolysing activity of HCWDEs. High temperature also prevents contamination by mesophilic microbes (Sarmiento *et al*. 2015). Moreover, CWDE‐inhibiting proteins, which are widely distributed in the plant cell wall as a defence mechanism (Benedetti *et al.,* 2018b; Juge 2006; Kalunke *et al.,* 2015; Locci *et al.,* 2019; York *et al.,* 2004), are also inactivated at high temperatures (Liu *et al.,* 2017; Locci *et al.,* 2019), thus do not interfere with enzymatic cellulose degradation. Furthermore, the structural stability of HCWDEs sustains their activity even in the presence of chemicals, surfactants and extreme pH (de Miguel Bouzas *et al*. 2006; Souza *et al*. 2016). Such harsh reaction conditions may be exploited in industrial applications to promote detachment of different lignocellulose components, thus further increasing the efficiency of HCWDE enzymatic hydrolysis (Li *et al*. 2016; Ooshima *et al*. 1986).

Algal productivity in both closed and open growth systems is affected by the competition of undesirable microorganisms. This problem has been tackled by metabolic engineering transforming the target organism with the gene encoding phosphite dehydrogenase D (PTXD) from *Pseudomonas stutzeri* WM88 (Loera‐Quezada, 2016; López‐Arredondo, 2012). As PTXD oxidizes PO_3_^3‐^ (phosphite) into PO_4_^3‐^ (phosphate), its expression confers the ability of metabolizing phosphite as the sole phosphorous source, allowing growth of the target organism in a phosphate‐depleted/phosphite‐repleted medium (Costas *et al*. 2001; Loera‐Quezada *et al*. 2016).

Here, we report the design and use of a transplastomic/transgenic *C*.*reinhardtii* expression system to produce a hyperthermostable cellulolytic blend, capable of breaking down polysaccharides of plant cell walls into simple sugars for fermentation. The hydrolysates from microalgal‐treated lignocellulosic materials were successfully used both to sustain mixotrophic growth of the microalga *C*.*vulgaris*, and to promote anaerobic digestion by methanogenic bacteria, allowing the establishment of a proof of concept for energy recovery from waste biomass (i.e. corn bran, corn cob). Moreover, the double‐transgenic microalgae (referred to as HC‐PTXD strain) were cultured in non‐sterile conditions using phosphite as the sole phosphorus source without loss in productivity. Notably, the microalgal extract showed a detoxifying effect towards the inhibitory by‐products released from the alkaline treatment of lignocellulosic biomass, opening the way to novel processing procedures for biofuel industry.

## Results

### Design of a *Chlamydomonas reinhardtii* biofactory for hyperthermophilic cellulases

We selected endoglucanase B (T‐EG) from *Thermotoga neapolitana* (Bok *et al*. 1998), the cellobiohydrolase portion of the CelB cellulosome (C‐CBH) from *Caldicellulosiruptor saccharolyticus* (formerly known as CBM3GH5, Park *et al*. 2011) and β‐glucosidase (P‐BG) from *Pyrococcus furiosus* (Kado *et al*. 2011; Kengen *et al*. 1993) as the hyperthermophilic cellulases (HCs) to be produced in *C*.* reinhardtii*. In addition, a hyperthermophilic xylanase (T‐XY) from *T*.* neapolitana* (Zverlov *et al*. 1996) was included as auxiliary enzyme (Hu *et al*. 2011; Figure S1A). Xylan is one of the most abundant types of hemicellulose, whose branched structure limits the access of cellulolytic enzymes to the cellulose component. Therefore, hemicellulose depolymerization is mandatory to efficiently degrade cellulose (Hayashi and Kaida, 2011, Benedetti *et al.,* 2019a). As efficient cellulose hydrolysis requires optimized enzymatic activities ratios, we decided to produce transgenic *C*.*reinhardtii* strains that express independently each of the four HCWDEs: it allows to optimize the enzymatic cocktail for maximum yield and to keep loading each activity as low as possible. Moreover, nuclear expression of *PTXD* gene from *Pseudomonas stutzeri* was implemented in HC‐producing strains to allow cultivation of *C*.* reinhardtii* in non‐axenic media without loss in algal productivity (Loera‐Quezada *et al*. 2016; López‐Arredondo and Herrera‐Estrella 2012; Figure S1B).

### Chloroplast expression of HCs in *Chlamydomona*s *reinhardtii*

Genes encoding the four components of the cellulolytic machinery (Figure S1) were codon‐optimized according to the codon usage of *C*.*reinhardtii* plastome and fused at the C‐terminus to a short HA‐epitope (i.e. 9 amino acids), which allows recombinant protein detection by immunoblot analysis. All these genes were cloned in the IR‐int vector (Day and Goldschmidt‐Clermont 2011; Faè *et al.,* 2017; Michelet *et al*. 2011) and independently introduced in *C*.*reinhardtii* by helium‐gun bombardment (Purton 2007). It is worth noting that the IR‐int vector targets the duplicated part of the chloroplast genome known as the inverted repeat (IR), so that in the transformed lines the transgene is present in two copies per genome and contains an *aadA* cassette that permits selection on media containing spectinomycin (Goldschmidt‐Clermont, 1991; Methods S1, S2).

Four independent spectinomycin‐resistant transformants expressing each enzyme were subjected to five consecutive rounds of selection until all the copies of the plastid genome contained the transgene (homoplasmy) (Faè *et al.,* 2017; Mayfield *et al.,* 2007). The absence of wild‐type copies of the plastome was confirmed by PCR using oligos annealing upstream and downstream of the homology regions used for the transgene integration (Figure S2). The genotyping of four putative C‐CBH expressing transformants, here reported as representative HC genotyping, revealed that only two out of four transformants were clearly homoplasmic (i.e. transformants #3 and #4, Figure S2). Notably, the two homoplasmic lines showed the highest C‐CBH activity that was comparable in both the transformants (Figure S3A). The dependence of the chloroplast‐expressed C‐CBH on temperature and pH showed it was characterized by a broader range of pH optimum than the recombinant version purified from *Escherichia coli* (Park *et al*. 2011), whereas the two enzymes showed a similar T optimum (Figure S3B‐C).

**Figure 1:**
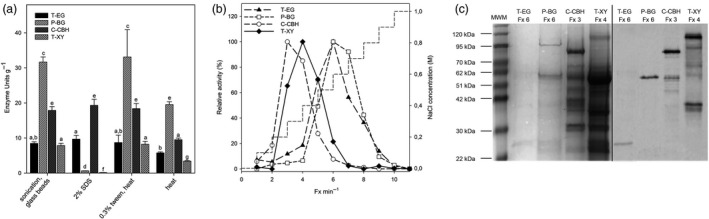
Chloroplast expression of HCs. (a) HC activity in extracts from *C*.* reinhardtii* cells, as obtained by either mechanical disruption (sonication + glass beads), denaturing conditions (2% SDS, RT), non‐denaturing conditions (0.3% Tween, 70°C) or with water and heat (70°C). See Method S3 for details. Data are expressed as mean ± SD, *n* = 3. Values marked with the same letters are not significantly different from each other (ANOVA test, *P* < 0.05). (b) The activity of HCs in the fractions (Fx) collected upon anion‐exchange chromatography, expressed as relative activity compared with the most enriched fraction. Profile of the elution gradient (NaCl concentration) is reported as short dash. (c) SDS‐PAGE and coomassie staining (*left panel*) and immunodecoration (*right panel*) analysis carried out on the fractions showing the highest activity. Molecular weights of the marker are indicated on the left.

To corroborate the presence, functionality and yield of each enzyme in cell extract of *C*.*reinhardtii*, immunodetection and enzymatic assays were performed. We first tested four different extraction methods: sonication in the presence of glass beads, heating at 70 °C, treatment with 2% SDS (w/v) and treatment with 0.3% Tween 20 (v/v) combined with heat treatment (Method S3). Heating at 70°C allowed extraction of the four HCs as demonstrated by enzyme activities (Figure 1a). Treatment with 2% SDS allowed for the extraction of active T‐EG and C‐CBH, whereas activity of P‐BG and T‐XY was extremely low, suggesting that P‐BG and T‐XY were sensitive to SDS treatment. An efficient extraction for the four HC enzymes was achieved by incubating cells with 0.3% Tween 20 (v/v) at 70°C or by mechanical destruction (sonication + glass beads). Similar enzyme activity levels were obtained for T‐EG (8.7 and 8.5 units/g DW), P‐BG (33.1 and 31.7 units/g DW), C‐CBH (18.4 and 17.9 units/g DW) and T‐XY (8.2 and 7.9 units/g DW) with both extraction methods (Figure 1a).

To determine specific activity and yield of each enzyme, a two‐step purification procedure, consisting of heat‐mediated enrichment (Patchett *et al*. 1989) followed by anion‐exchange chromatography (AEC), was implemented (Method S4). The selected HCs, which strongly bind to the AEC column because of their acidic pI, were eluted at different NaCl concentrations ranging from 0.3 to 0.6 m. Enzyme activity of the recovered fractions was used to track the enzymes upon AEC (Figure 1b). Subsequently, the fractions with the highest activity were further evaluated by SDS‐PAGE analysis and immunoblot to detect the corresponding enzymes. Notably, for each HC, bands of the expected molecular weight were detected by SDS‐PAGE analysis and confirmed by immunodecoration analysis (Figure 1c) as demonstrated in previous experiments using total cell extracts (Figure S4). Determining enzyme concentration allowed the calculation of the specific activity of each protein (expressed as enzyme units per mg enzyme, Table S1), which, in turn, was used to estimate the amount of each enzyme in the raw cell extract. The highest yield was obtained for C‐CBH (0.8–1 mg/g DW alga), followed by P‐BG (0.3–0.4 mg/g DW alga) and T‐XY (0.2–0.3 mg/g DW alga). In the case of T‐EG, we observed a low yield (0.02–0.03 mg/g DW alga), which was consistent with the weak immunoblot signal observed in the corresponding cell extracts (Figure S4). The abundance of these enzymes spanned a range from 0.015% (T‐EG) to 0.5% (C‐CBH) of the total soluble proteins (TSP) in algal cells.

### HC‐producing lines grow using phosphite as the sole phosphorus source

HC‐producing strains were metabolically engineered to use phosphite as the sole phosphorous source, by nuclear expression of the *PTXD* gene from *P*.* stutzeri* (Loera‐Quezada *et al*. 2016; López‐Arredondo and Herrera‐Estrella 2012). The *PTXD* gene was introduced in each HC‐producing strain by electroporation to obtain the four HC‐PTXD strains (Method S2). The plasmid pChlamy4‐PTXD (Loera‐Quezada *et al*. 2016) contained the sequence encoding the foot‐and‐mouth disease virus 2A self‐cleavage peptide to transcriptionally fuse the PTXD expression to zeocin resistance (Rasala *et al*. 2012). We obtained a total of 145 independent zeocin‐resistant clones, from which four (i.e. one for each HC‐PTXD strain) were selected for efficient grow in a medium containing up to 4 mm potassium phosphite (KH_2_PO_3_) as the sole phosphorus source (Figure S5). This result shows that chloroplast transformation for the expression of recombinant protein was compatible with a nuclear transformation allowing the growth on a selective media using phosphite to control the growth of contaminant organisms.

### Optimization of HC chloroplast expression in the double HC‐PTXD transformants

**Figure 2:**
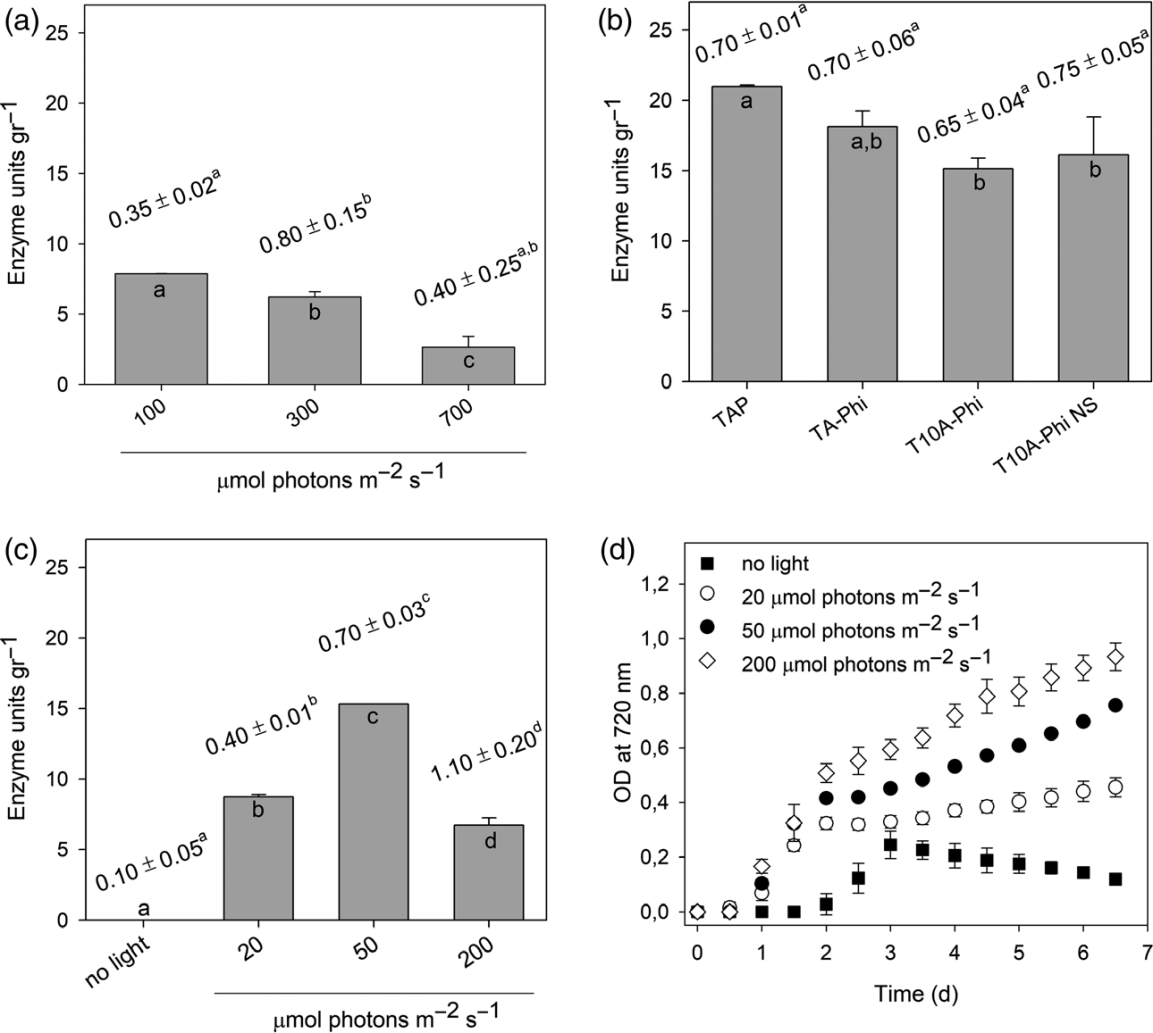
Optimization of C‐CBH chloroplast expression. (a) Enzyme activity measured in extracts from cultures grown under photoautotrophic condition at three different irradiances. (b) Enzyme activity determined in extracts from cultures grown under mixotrophic conditions, at 50 µmol photons/m^2^/s and different growth media: TAP, TA‐Phi (TAP medium in which K_2_HPO_4_ was replaced by 1 mm KH_2_PO_3_), T10A‐Phi (TA‐Phi with lower Tris content) and T10A‐Phi NS (unsterile T10A‐Phi medium). (c) Enzyme activity measured in extracts from cultures grown in T10A‐Phi NS medium under different light irradiances. Numbers above each column indicate the biomass produced (g DW/L) after 7 days of growth. Values are reported as mean ± SD, *n* = 3, and are representative of two independent biological replicates. For each parameter quantified, values marked with the same letters are not significantly different from each other (ANOVA test, *P* < 0.05). (d) Growth curves of the cultures described in (c) followed by recording OD at 720 nm. 2.5 × 10^5^ cell/mL was used as starting inoculum. Statistical analysis (F test) revealed that algal culture maintained at 200 µmol photons/m^2^/s grew significantly more than all other cultures (*P* < 0.05). Experiments were repeated twice with consistent results.

To achieve the highest production levels of the chloroplast recombinant enzymes, the establishment of optimal growth conditions is desirable. As a representative of the system, we assessed the production of C‐CBH per unit of alga biomass by the double transformant C‐CBH‐PTXD in different growth media and light conditions seven days after inoculation (Figure 2a‐c; Method S5). Line 3 harbouring C‐CBH and PTXD was subjected to photoautotrophic conditions by growth in medium without a carbon source (HS medium), under three different light intensities (100, 300 and 700 µmol photons/m^2^/s). Production level of C‐CBH under the three different light conditions was very low, ranging from 3 to 7.5 enzyme units/g DW. However, biomass production was higher under 300 µmol photons/m^2^/s achieving 0.8 g DW/L, as compared to 0.35 and 0.4 g DW/L achieved under 100 and 700 µmol photons/m^2^/s, respectively (Figure 2a). We then tested whether mixotrophic conditions enhance the yield of C‐CBH. For this purpose, cultures using standard TAP media under 50 µmol/m^2^/s light intensity were grown. Both biomass production and enzyme accumulation were increased under this condition, allowing the production of 0.7 g DW/L in one week and more than 20 enzyme units/g DW (Figure 2b). These results suggest by supplying an external carbon source we could increase the growth of *C*.* reinhardtii* and the recombinant enzyme expression. The same conditions were applied to produce T‐EG, P‐BG and T‐XY.

In order to test whether the phosphite‐based system allows the production of C‐CBH in *C*.* reinhardtii*, we performed experiments to evaluate growth of the double C‐CBH‐PTXD transformant and enzyme production in a modified version of the TAP media supplemented with phosphite as the sole phosphorus source. In order to reduce the production cost of culturing *C*.* reinhardtii*, a cheaper growth medium was developed. The new medium, named as T10A‐Phi, contained only 10% (0.24 g/L) of the amount of Tris used in conventional TAP media (Kropat *et al*. 2011). The C‐CBH‐PTXD transformant was cultured in T10A‐Phi media supplemented with 1 mm Phi under 50 µmol photons/m^2^/s light intensity under sterile and non‐sterile conditions, and enzyme production was assessed seven days after inoculation. The double C‐CBH‐PTXD transformant showed vigorous growth in the TA‐Phi media and produced similar biomass amount as when grown using the TAP standard media (Figure 2b). However, enzyme units' yield showed a slight reduction (13%) as compared to TAP media. In the low‐cost T10A‐Phi media, growth of the double transformant under sterile and non‐sterile conditions was also vigorous with no statistical difference in comparison with the TA‐Phi control medium (Figure 2b). Importantly, growth of the C‐CBH‐PTXD transformant under non‐axenic media and non‐sterile systems did not significantly affect the yield of C‐CBH nor the growth of the strain (Figure 2b).

In order to assess the best light intensity for growth of the C‐CBH‐PTXD strain under mixotrophic conditions using phosphite as phosphorus source, we measured the growth and enzyme production with three different light conditions (20, 50 and 200 µmol photons/m^2^/s) and darkness. Interestingly, cultivation in T10A‐Phi medium with no light strongly affected *C*.* reinhardtii* cultivation as the transformant showed a stunted growth (Figure 2c, d). Growth under 20 µmol photons/m^2^/s rendered the lowest biomass concentration (0.4 g DW/L), 50 µmol photons/m^2^/s produced an intermediate amount of biomass (0.7 g DW/L), whereas 200 µmol photons/m^2^/s produced the highest level of biomass (1.2 g DW/L; Figure 2c, d). However, under 200 µmol photons/m^2^/s C‐CBH production was reduced to below 10 enzyme units/g. Cultivated under 50 µmol photons/m^2^/s, the algae were producing 10.7 U/L, which was higher when compared to the culture at 200 µmol photons/m^2^/s, 7.7 U/L (Figure 2c, d). This suggests 50 µmol photons/m^2^/s as the optimal light intensity for expressing C‐CBH in *C*.* reinhardtii* under mixotrophic conditions.

### The HC‐PTXD algal mixture presents efficient hydrolysing activity against cellulosic substrates and is stable under prolonged high‐temperature conditions

**Figure 3:**
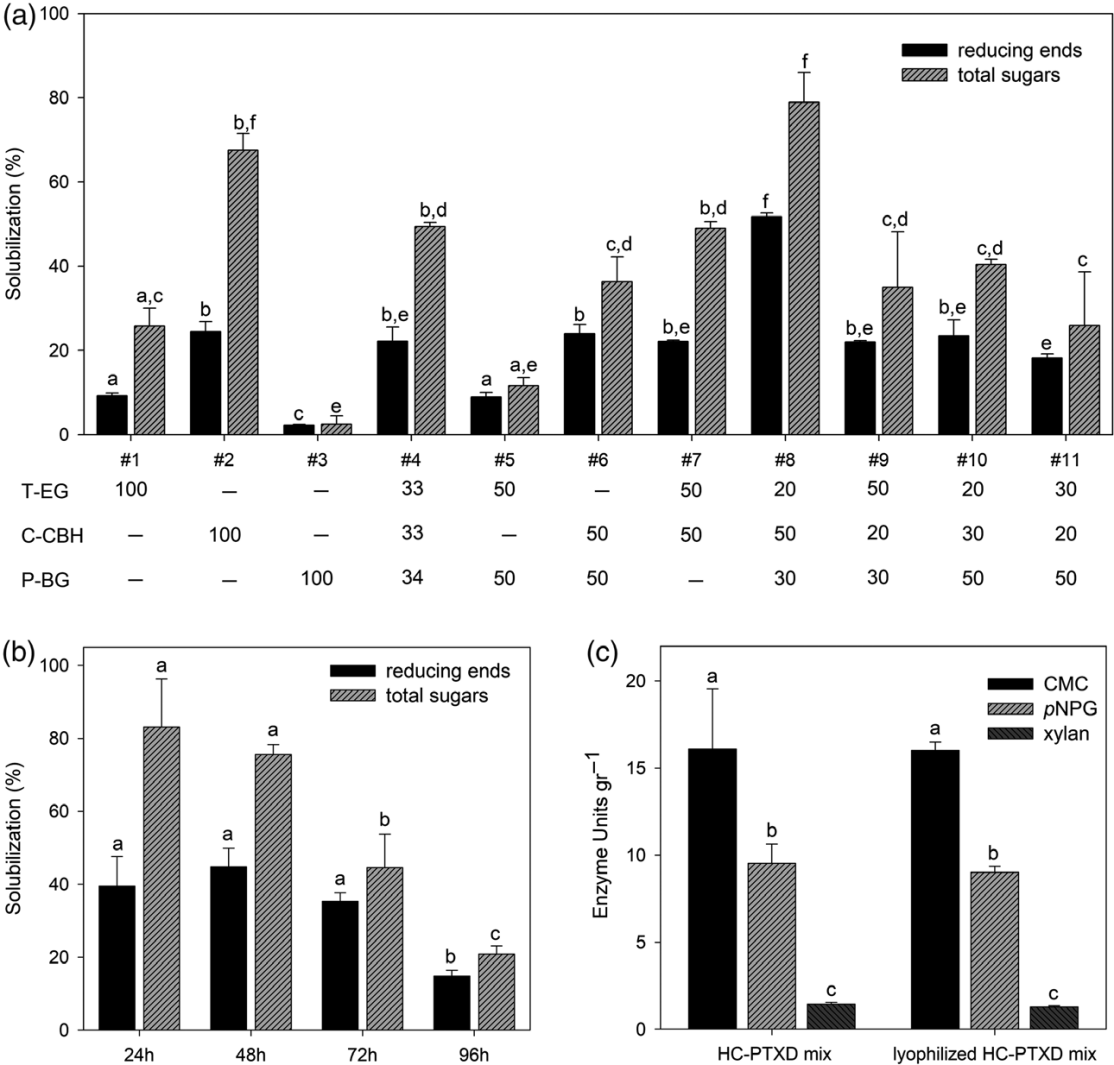
Determination of the optimal composition of HC‐PTXD mixture. (a) Solubilization of PASC into reducing ends (black bar) and sugars (striped bar) by the extracts from different HC‐PTXD mixtures (#1‐11). Numbers underneath indicate the percentage of the corresponding HC‐PTXD strain (w/w) used in each combination. (b) Enzymatic stability of the extract from HC‐PTXD mix #8. Solubilization of PASC into reducing ends (black bar) and sugars (striped bar) was measured upon incubation with the extract from HC‐PTXD mix #8. At the end of each 24‐h cycle, insolubilized cellulose was removed and fresh PASC was added to the reaction mixture, for a total of four 24‐h cycles. (c) Activity of HC‐PTXD mix #8, supplemented with T‐XY, measured towards CMC (black bar), *p*NPG (striped bar) and xylan (dark striped bar). The analysis was performed before (HC‐PTXD mix) and after drying and storage of the algal biomass at 22°C for 1 month (lyophilized HC‐PTXD mix). Data are expressed as mean ± SD, *n* = 3. For each parameter quantified, values marked with the same letters are not significantly different from each other (ANOVA test, *P* < 0.05).

In order to determine the optimal composition of a microalgae‐based enzyme blend with the high cellulose‐degrading activity, we prepared 11 different cocktails by mixing dry biomass from three HC‐PTXD lines in variable proportions (T‐EG:C‐CBH:P‐BG) (w/w/w). Upon non‐denaturing extraction treatment, the supernatant was collected and enzyme activity and catalytic stability of the different HC combinations were evaluated (Figure 3). It is worth noting that all HCs had optimal activity in a pH range between 5 and 6 (Bok *et al*. 1998; Kengen *et al*. 1993; Park *et al*. 2011; Zverlov *et al*. 1996). Hydrolysing activity of different enzyme cocktails was assayed towards phosphoric acid swollen cellulose (PASC) upon 1‐day incubation at 75°C (Method S6). These conditions allow the determination of reducing ends and total sugars produced for each mixture of the different recombinant strains (Method S7). The extract from mixture #8 (T‐EG:C‐CBH:P‐BG 20:50:30, w/w/w) solubilized PASC more efficiently (80%) than any other combination tested and produced the highest amount of reducing and total sugars (Figure 3a). Mixture #8 (hereafter called HC‐PTXD mix), composed by a ratio of dry algal powder of 20:50:30 (T‐EG:C‐CBH:P‐BG; w/w/w), is characterized by a large abundance of C‐CBH, which is similar to the composition of degradative secretions produced by cellulolytic fungi such as *Trichoderma viridae,* where cellobiohydrolases account for up to 70% of total glycosyl hydrolases (Brady *et al*. 2015; Teeri 1997). Consistently, the CBH content in this mixture was 66% C‐CBH (0.5 mg/g DW), 5% T‐EG (0.01 mg/g DW) and 29% P‐BG (0.15 mg/g DW).

To assess the thermostability of the HC‐PTXD mix, enzymatic activity of the combined algal powders was tested under prolonged high‐temperature treatment. In particular, the ability of the enzyme cocktail to trigger sugar release from PASC was quantified every 24 h and concomitantly fresh PASC was supplemented to the reaction mix after each cycle of reaction (24 h/cycle). PASC hydrolysation, expressed as percentage of solubilization, was comparable during the first two cycles with no statistical difference in reducing and total sugars released (Figure 3b), thus indicating that the enzyme cocktail remained stable for at least 48 h at 70°C. However, during the third (72 h) and fourth (96 h) cycles, a decrease of ~50% and ~70%, respectively, in total sugars released was detected in comparison with the first cycle. Interestingly, the ratio of reducing ends: total sugars increased as the reaction time increased, suggesting a gradual reduction in both endoglucanase and cellobiohydrolase activities. This decrease in enzyme activities can be due to a gradual inactivation of T‐EG and C‐CBH due to protein denaturation or enzymatic inhibition mechanisms.

To evaluate the hydrolytic potential of the HC‐PTXD mix towards more complex substrates such as the lignocellulosic material, we supplemented the enzyme blend with T‐XY in a 80:20 proportion (HC‐PTXD mix:T‐XY, w/w) since degradation of xylans is highly effective in boosting the activity of cellulolytic enzymes (Benedetti *et al.,* 2019a). To assess the long‐term stability at room temperature (RT, 22°C) of enzyme activities in the HC‐PTXD mix for different processes, we determined its hydrolytic activity on different substrates upon long‐term storage. Enzymatic activity was assayed towards different substrates such as carboxymethylcellulose (CMC) to determine cellulase activities, *p*‐nitrophenyl‐β‐glucopyranoside (*p*NPG) to determine β‐glucosidase activity and xylan from beechwood to determine xylanase activity. Interestingly, similar hydrolytic efficiencies were obtained for the enzymatic mixture, before and after storage at RT for 1 month. These results suggest that storage of the dry powder for 1 month at RT does not have a negative effect on the activity of the various HCs present in the HC‐PTXD mix (Figure 3c). Interestingly, analysis of substrate specificity towards different cellulosic substrates including PASC, CMC and Avicel revealed that the HC‐PTXD mix displayed also detectable activity towards microcrystalline cellulose (Avicel PH101), although cellulolytic oxidoreductases were missing in the mixture (Table S2).

### **Application of PASC hydrolysis products both enhances the biomethane potential of anaerobic digestion and supports the mixotrophic growth of *C***. ***vulgaris***

**Figure 4:**
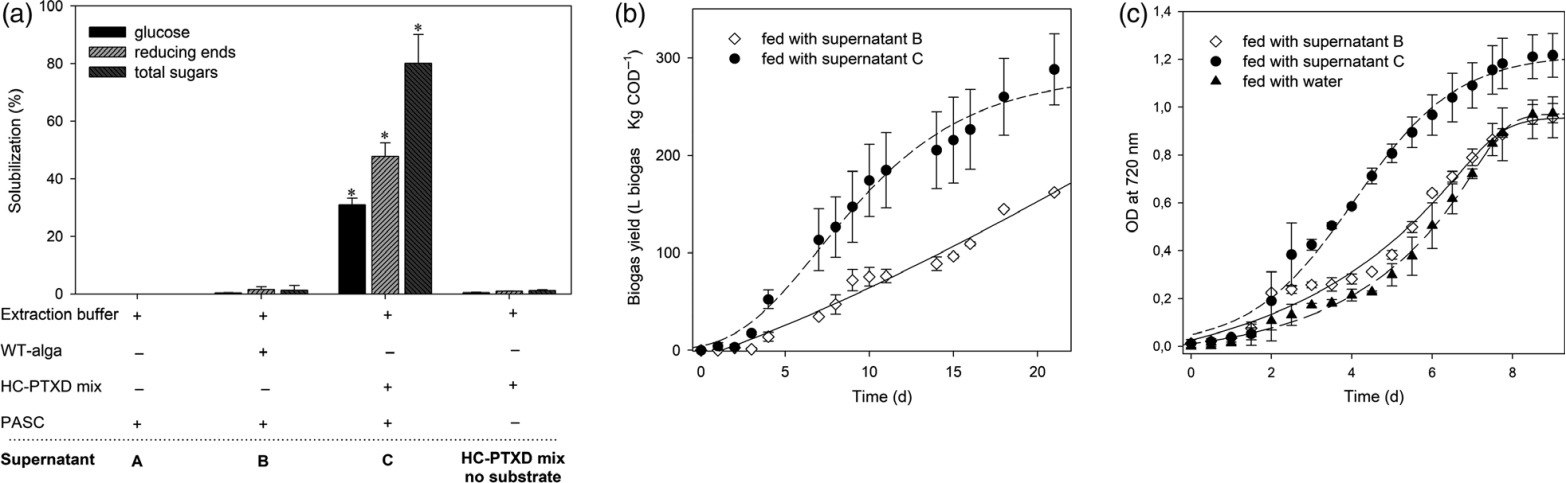
Application of algal‐treated PASC both enhances biogas yield and supports the mixotrophic growth of *C*.*vulgaris*. (a) Solubilization of PASC (0.6% w/v) into glucose (black bar), reducing ends (striped bar) and sugars (dark striped bar) after incubation with either the extraction buffer (supernatant A), with the extracts from wild‐type microalga (supernatant B) or from HC‐PTXD mix (supernatant C). For each parameter quantified (glucose, reducing ends, total sugars), treatment with HC‐PTXD mix resulted in significantly higher PASC solubilization (*) than treatment with extract from WT algae (Student’s *t‐*test, *P* < 0.05). A solubilization trial including HC‐PTXD mix without substrate is reported as negative control. (b) Production of biogas (L Kg/COD) from methanogenic bacteria suspension from digested sludge, fed with 200 mL/L of the supernatant B or C. Biogas yields were corrected according to the control reaction (production of biogas without adding supernatants). (C) Growth curves of *C*.*vulgaris* fed with either water or 140 mL/L of the supernatant B or C. Starting inoculum of *C*.*vulgaris* was 2.5∙10^5^ cell/mL. Growth was followed by recording OD at 720 nm. Data are reported as mean ± SD, *n* = 3. Statistical analysis (*F* test) revealed that algal culture fed with supernatant C grew significantly more than all other cultures (*P* < 0.05). Experiments were repeated twice with consistent results.

Having demonstrated that the HC‐PTXD mix presents an efficient hydrolysing activity against PASC, the hydrolysates were used to study their potential to sustain biomethane production by anaerobic digestion and to support the mixotrophic growth of *Chlorella vulgaris*, a microalgae of interest for industrial‐scale production of biofuels (Liu and Chen 2016; Mallick *et al*. 2016; Method S8, S9). Both methanogenic bacteria and *C*.*vulgaris* are able to metabolize monosaccharides, several disaccharides (including cellobiose) and short‐chain oligosaccharides (Dvořáková‐Hladká 1966), and therefore are good candidates to be cultivated on the products of PASC hydrolysis. We measured the amount of reducing and total sugars in the supernatant of PASC treated with the enzyme cocktail (Figure 4, supernatant C). We used an extract of wild‐type *C*.*reinhardtii* (WT alga, supernatant B) and the extraction buffer alone (supernatant A) as a control. The extract from HC‐PTXD mix degraded most of the cellulose (80%), although the low ratio [glucose: reducing ends: total sugars] indicated that the solubilized cellodextrins were not efficiently converted into glucose (Figure 4a). Consistently, cellulose was converted into glucose with higher efficiency (75‐90%, see Figure S6) at a lower substrate concentration (i.e. 0.3% w/v). As expected, treatments of PASC with either the incubation buffer only or the extract from the *C*.*reinhardtii* wild‐type strain failed to hydrolyse cellulose (Figure 4; Figure S6). To test the potential of the hydrolysates to sustain biomethane production and *C*.*vulgaris* growth, the supernatants from PASC treated with wild‐type and HC‐PTXD extracts (supernatants B and C) were administered to either an inoculating sludge of methanogenic bacteria or a *C*.*vulgaris* culture in BG‐11 medium. Methanogenic bacteria were monitored for biogas production (expressed as L net biogas kg/COD) and *C*.*vulgaris* for growth under mixotrophic conditions. Since microalgal biomass is a valuable feedstock for biogas production (Figure S7), treatment of cellulosic substrates was performed by using the clarified supernatant cleared of the cell debris, also a supernatant from wild‐type algae was used as a control in order to avoid a possible bias in the interpretation due to the bio‐stimulant action of algal biomass (Montingelli *et al*. 2015; Mussgnug *et al*. 2010). After 22 days of anaerobic digestion, methanogenic bacteria fed with the supernatant obtained from PASC treated with HC‐PTXD mix extract rendered 80% higher biogas than the amount produced by the sample fed with the supernatant obtained from PASC treated with wild‐type alga (Figure 4b). Overall, these results emphasize the effect of the HC‐PTXD mix on significantly enhancing the hydrolytic phase in the first days of reaction, a fundamental aspect to reduce the retention time of anaerobic digestion reactors or to reduce their volume.

Alternatively, the PASC supernatants (Figure 4a) were used for feeding *C*.*vulgaris* to study their potential to support its mixotrophic growth. With this purpose, the same supernatants were supplemented to *C*.*vulgaris* culture in BG‐11 medium and OD_720_ monitored for nine days. Addition of supernatant from the PASC treated with wild‐type microalgal extract (supernatant B) did not show any growth enhancement over the control sample fed with water. Instead, the final biomass yield of *C*.*vulgaris* was significantly higher when the culture was fed with hydrolysates from the HC‐PTXD‐treated PASC (supernatant C), than with water and the wild‐type hydrolysate (Figure 4c). Accordingly, the HC‐PTXD mix extract devoid of the substrate contained negligible amount of sugars (Figure 4a) and did not affect *C*.*vulgaris* growth (Figure S8). Taken together, these results suggest that PASC hydrolysates produced by the HC‐PTXD mix represent a source of nutrients to support *C*.*vulgaris* growth and can be used as a substrate by methanogenic bacteria. Therefore, HC‐PTXD mix could have potential to be used as part of the biological treatment for the conversion of plant biomass to produce biofuels.

### The HC‐PTXD mix efficiently hydrolyses chemical‐treated corn cob flour and corn bran

Residual lignocellulose from corn (*Zea mays*), for example cob flour and bran, represents one of the most abundant agro‐industrial wastes as well as a potential feedstock for biological conversion towards renewable chemicals (Gibreel *et al*. 2009; Zheng *et al*. 2014). Corn cob is composed of 42% (w/w) of cellulose, 33% of hemicellulose and 18% of lignin, and it roughly represents 15%–20% of the total agricultural residues from corn material (Schwietzke *et al*. 2009). Production yield of corn bran is about 60–70 g kg/DW corn kernels (Watson *et al.,* 2003) accounting for 3%–4% of the plant biomass. Corn bran is composed of 28%–30% of cellulose, 55%–65% of hemicellulose and lignin and other phenolic compounds for less than 5%–7% (Rose *et al*. 2010). We tested the potential of microalgal‐produced cellulolytic enzymes to degrade cellulosic scraps from corn cob flour and corn bran. To enhance the deconstruction of the polysaccharide matrix present in these complex lignocellulose biomasses, pretreatments with diluted acid, alkali and/or steam explosion are commonly used (Kumar and Sharma, 2017). Therefore, we first evaluated different physical and chemical methods to determine the pretreatment best suited for corn cob flour (Figure S9; Method S10). To test the potential of the HC‐PTXD mix extract to increase solubilization of the residual material after pretreatment with chemicals and heat, we added algal extracts from the WT (WT alga) and the HC‐PTXD mix, and then measured the level of solubilization after one day of incubation. As expected, no increase in cellulose solubilization was observed when pretreated materials were incubated with WT extract. Conversely, incubation with the HC‐PTXD mix extract resulted in an increase of the solubilization level between 20% and 30% after both treatments with sulphuric acid. Alkaline treatment boosted enzymatic degradation only in biomass pretreated by the thermic treatment (TT/AK‐TT) with an increase of 30% solubilization (Figure S9B). These results suggest that the TT/AK‐TT treatment of raw biomass represents the best compromise between biomass recovery after pretreatment (Figure S9A) and the hydrolysing efficiency by the HC‐PTXD mix (Figure S9B). Using an enzyme:substrate ratio similar to that employed in the treatment of PASC (1 g HC‐PTXD mix: 1.3 g TT/AK‐TT corn biomass), we performed a time‐course analysis following glucose, reducing ends and total sugars released from TT/AK‐TT corn cob flour incubated with a HC‐PTXD mix. We observed that the enzyme cocktail was able to degrade the complex lignocellulosic biomass, reaching a plateau after 48h of incubation without further production of soluble sugars (Figure S10). Considering that HC‐PTXD mix contained about 0.65 mg enzyme blend g/DW, the final enzyme concentration used in degrading 1.3 g TT/AK‐TT corn biomass was 0.05% (w/w).

### Corn cob flour and corn bran provide a valuable source of material for biofuel production by HC‐PTXD mix hydrolysis

**Figure 5:**
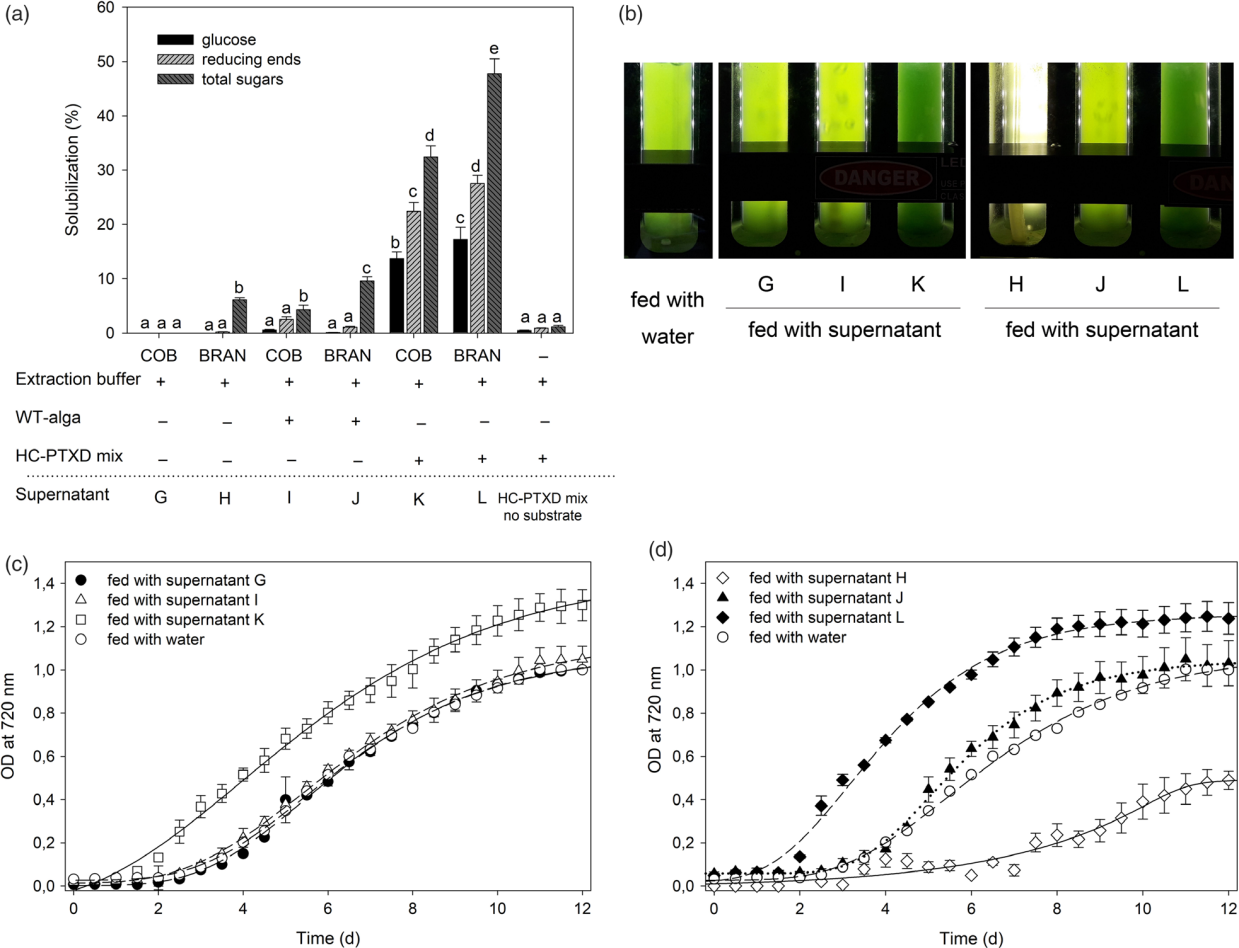
Mixotrophic growth in *C*.*vulgaris* by using algal‐treated agro‐industrial wastes as feed. (a) Solubilization of TT/AK‐TT corn cob flour and corn bran (0.8% w/v) into glucose (black bar), reducing ends (striped bar) and sugars (dark striped bar) was measured upon incubation with either the extraction buffer only (supernatants G, H), with the extracts from wild‐type microalga (supernatants I, J) or the extracts from HC‐PTXD mix (supernatants K, L). Values marked with the same letters are not significantly different from each other (ANOVA test, *P* < 0.05). A solubilization trial including HC‐PTXD mix without substrate is reported as negative control. (b) A representative picture of *C*.*vulgaris* cultures described in (a), after 8 days of growth. A culture fed with water is reported as negative control. (c, d) Growth curves of the same *C*.*vulgaris* cultures described in (b), followed by recording OD at 720 nm. Cultures were fed with supernatants from treated TT/AK‐TT corn cob (c) or corn bran (d). 2.5 × 10^5^ cells/mL was used as starting inoculum. Data are reported as mean ± SD, *n* = 3. Statistical analysis (*F* test) revealed that algal cultures fed with supernatants K and L grew significantly more than cultures fed with water (*P* < 0.02) and that culture fed with supernatant H was significantly retarded in growth than culture fed with water (*P* < 0.01). Experiments were repeated twice with consistent results.

As previously shown with the PASC hydrolysates, soluble sugars obtained from the degradation of cellulose can sustain the mixotrophic growth of *C*.*vulgaris*. Therefore, we explored the effect of the supplementation of corn cob flour and corn bran hydrolysates, obtained as previously described, to the same microalga. With this aim, TT/AK‐TT cob flour and bran were separately incubated with extracts from the WT alga (supernatants I and J), the HC‐PTXD mix (supernatants K and L) or extraction buffer alone (supernatants G and H), and the amount of hydrolysed sugars determined after two days of reaction (Figure 5). Low or no solubilization was observed when buffer alone or WT alga extract was added to either of the cellulosic materials. Importantly, the HC‐PTXD mix enzyme cocktail was capable of efficiently releasing soluble sugars from both TT/AK‐TT cob flour and bran. In particular, HC‐PTXD mix converted the TT/AK‐TT cob flour and bran into glucose with efficiencies of 14% and 17%, respectively, thus indicating that TT/AK‐TT bran was a better source of cellulosic material (Figure 5a). This may be attributed to the higher starting lignin content of cob *vs*. bran (Chen *et al*. 2013; Rose *et al*. 2010; Schwietzke *et al.,* 2009). The hydrolysate supernatants obtained from TT/AK‐TT corn materials (G, H, I, J, K, L) were then used as a carbon source to promote *C*.*vulgaris* biomass production. In this case, the supernatants obtained by incubating the extraction buffer with the TT/AK‐TT corn material were included as control‐feed since chemical‐treated lignocellulose may release inhibitory by‐products that inhibit microbial and enzymatic biocatalysts (Jönsson and Martín 2016). The growth of *C*.*vulgaris* in BG‐11 media was significantly enhanced when supplemented with hydrolysates produced from both corn cob flour and bran treated with TT/AK‐TT and HC‐PTX mix (Figure 5b‐d) respect to culture media supplemented with supernatants from corn cob treated with the WT algal extract, water or extraction buffer (Figure 5b, c; Method S11). However, the *C*.*vulgaris* culture fed with the incubation buffer from TT/AK‐TT bran (supernatant H; Figure 5b, d) grew slower and its biomass yield was strongly reduced as compared to those fed with either water or incubation buffer from TT/AK‐TT cob (supernatant G; Figure 5b, c, Figure S11), suggesting that inhibitory by‐products were released from TT/AK‐TT bran under thermal/alkaline treatment. Interestingly, cell extracts from *C*.* reinhardtii* abolished such inhibitory effect and restored the growth rate to the level obtained with *C*.* vulgaris* in control conditions (Figure 5b, d). Importantly, the biomass productivity of *C*.* vulgaris* was enhanced by 20 and 70% when HC‐PTXD mix was used to treat corn cob and bran, respectively, as compared to the control treatment, similar to what observed in *Chlorella* cultures fed with HC‐PTXD‐treated PASC (Figure 4c). In fact, this is consistent with both hydrolysates having a similar sugar content, 0.4% (w/v) in the treated PASC and 0.3% (w/v) in the treated corn material.

## Discussion

The development of an enzymatic preparation for the biological deconstruction of lignocellulosic biomass is still a major challenge for biofuel production. To achieve an efficient conversion of lignocellulosic residues into fermentable sugars, current enzyme‐based products require pretreatment of lignocellulosic material by harsh physical and chemical methods including steam explosion (Agbor *et al*. 2011; Mosier *et al*. 2005), wet oxidation (Varga *et al*. 2003), the use of ionic liquids (Prado *et al*. 2012), alkaline (Xu *et al*. 2016) or dilute acid treatments (Kumar and Sharma 2017). Therefore, there is urgent need for replacing polluting methods with environmentally friendly enzyme‐based strategies to make feasible the production of biofuels from lignocellulosic residues. To date, enzymatic hydrolysis of lignocellulosic agro‐industrial scraps is characterized by low efficiency and high operating costs, essentially due to (i) the incomplete knowledge of the enzymatic activities required by the process (Alessi *et al*. 2017), (ii) the limited efficiency of microbes to degrade raw lignocellulosic materials and (iii) the low expression levels of CWDEs which impact the costs of the microbial‐derived products with (thermolabile) cellulolytic activities (Herrero‐Garcia *et al*. 2019).

Plant expression of CWDEs is an interesting alternative to microbial‐based biofactories because of their high productivity/production cost ratio and carbon‐neutral process. However, expression of CWDEs in plants could have side effects as CWDEs from lignocellulolytic fungi and bacteria are well‐known pathogenic factors that could have detrimental effects for plant growth (Benedetti *et al*. 2019b; Choi and Klessig, 2016; Ma *et al*. 2015; Poinssot *et al*. 2003). To prevent potential side effects of the expression of CWDEs in plants, different strategies have been proposed such as compartmentalized expression/accumulation (Park *et al*. 2016), inducible gene expression (Tomassetti *et al*. 2015) and expression of CWDEs with inducible activity, for example hyperthermophilic enzymes (Mir *et al*. 2017). Compartmentalized expression of CWDEs in the chloroplast may enhance the yield of recombinant protein since chloroplast expression is not prone to gene silencing (Li *et al*. 2019); nevertheless, the use of endogenous promoters and other cis‐acting elements to drive chloroplast expression of transgenes may be subjected to the host regulation, requiring the optimization of specific growth conditions in order to enhance recombinant protein yield (Fields *et al*. 2018, Figure 2). Transplastomic tobacco plants that accumulate high level of GH5, GH6 and GH9 endoglucanases and pectin lyases have been reported (Faè *et al*. 2017; Schmidt *et al*. 2019; Verma *et al*. 2010). Moreover, transplastomic tobacco plants expressing the GH3 β‐glucosidase Bgl1 from *Trichoderma reesei* had an increased biomass yield and an improved resistance towards aphids than wild‐type plants (Jin *et al*. 2011). Expression of GH10 xylanase from *Alicyclobacillus acidocaldarius* (Xyn) (Castiglia *et al*. 2016) produced both healthy plants and high enzyme activity (Table 1), whereas plant treatment with GH11 xylanases from different fungal pathogens induced an immune reaction in plants, independently of their enzymatic activity, thus pointing to the CWDE–plant interaction as strictly dependent on the specific CWDE (Frias *et al*. 2019). Nevertheless, according to recent results, the yield of chloroplast‐expressed cellulases is higher in transplastomic tobacco plants than in microalgae (Table 1).

Here, we report on a novel strategy to use *C*.* reinhardtii* as an efficient biofactory to produce CWDEs in a more cost‐effective manner that could make it an alternative to plant‐based systems. *C*.* reinhardtii* can grow at a much faster rate than plants, have an immune system less prone to react to plant CWDEs and reach higher productivities on cheap substrates (Specht *et al*. 2010). Moreover, culturing microalgae in non‐axenic conditions, by phosphite selective nutrition, may significantly reduce the current cost of algal biomass production. The combined use of (i) PTXD together with (ii) a cheaper growth medium (i.e.T10APhi medium) and (iii) a low‐light demand for optimal CWDE‐expression (50–100 µE, Figure 2b‐c) can reduce production cost of PBR‐grown microalgae to 3.2–3.8 € kg/DW as argued by Tredici *et al*. (2016) and Slade and Bauen (2013), a value close to that reported for field‐grown tobacco plants (2 € kg/DW; Maksymowicz, and Palmer 1997; Schmidt *et al*. 2019). Similarly, transgenic tobacco production is low cost in open field and under optimal conditions, which unfortunately could only be achieved in countries with no restriction on GMO cultivation, while production cost is significantly higher for greenhouse‐grown plants (6 € kg/DW; Faè *et al*. 2017). Moreover, microalgae are still more productive than an optimal tobacco production system with 3 growth cycles per year (60 t DW/ha/y *vs* 8.1 t DW/ha/y) (Giovannoni *et al*. 2020). However, two important aspects still need to be addressed to enhance potential for production of HCWDEs: (i) HC‐PTXD microalgae expressed HCWDEs at variable levels, ranging from 0.003% to 0.1% (w/w), pointing to the need of genetic strategies to optimize transgene expression, and (ii) the highly diverse level of expression we obtained for T‐EG, C‐CBH, P‐BG and T‐XY, pointed to the importance of ensuring sufficient level of enzyme catalysing the rate‐limiting step for the overall process in the blend. The high enzyme cost from *C*.* reinhardtii*‐based biofactory suggests that the microalgal system might be economically competitive over *N*.* tabacum* when producing those categories of CWDEs whose expression might be challenging in tobacco plants (Table 1). The use of HCWDEs has several advantages, mainly deriving from the high temperature at which these enzymes are active (Unsworth *et al*. 2007). High temperature loosens plant cell wall structures compensating the low functional heterogeneity of the HC‐PTXD mix and prevents most microbial contaminations, thus increasing the yield of saccharification. Moreover, HCWDEs are characterized by robust structures that confer marked enzymatic stability: the HC‐PTXD mix can be stored in the form of dried powder at RT for a long time without significant loss of activity (Figure 3c), while some of the blend components (i.e. T‐EG and C‐CBH) proved to be resistant to 2% SDS (Figure 1a), thus pointing to a possible exploitation under severe reaction conditions. Moreover, the robustness of HCWDEs also allowed the possibility of recycling the HC‐PTXD mix for three consecutive 24h‐reaction cycles (Figure 3b), thus reducing the enzyme loading in the process.

In our instance, the use of a thermostable (GH12/GH5/GH1)‐based blend allowed the conversion of alkaline‐treated lignocellulose into glucose with efficiencies ranging from 14% to 17% upon 48 h of reaction and an enzyme loading of 0.05% (w/w) (Figure 5a). Notably, the HC‐PTXD mix is further valorized by some intrinsic characteristics of the algal extract: that is the biogas stimulating property (Figure 4b, Figure S7) and the detoxifying action towards the inhibitory by‐products released from lignocellulosic biomass upon alkaline pretreatment (Figure 5b, d), a well‐recognized problem in cellulose degradation (Jönsson *et al*. 2013; Jönsson and Martín 2016), that may synergistically act with the above listed enzymatic features. At present, the commercially available enzymatic blends (including last‐generation enzyme‐based products, e.g. Cellic CTec3 for hydrolysis of lignocellulosic materials, Novozymes, Denmark) still require biomass pretreated by physico‐chemical methods for an efficient hydrolysis.

Chloroplast expression in *N*.* tabacum* offers the advantage of bio‐containing transplastomic plants *in situ* since the plastome is maternally inherited, and therefore cannot be dispersed through the pollen by vertical gene transfer (Daniell 2007). However, in the case of *C*.* reinhardtii* as a biofactory to produce CWDEs, biocontainment strategies would be necessary to avoid its accidental dispersion to the environment. Some technologies that could be combined with the CWDE‐expressing Chlamydomonas is the CRISPR‐Cas9 technology, developed by Baek *et al*. (2016), that can be used to generate nitrate reductase‐deficient *C*.*reinhardtii* strains, capable of only surviving in ${\text{NO}}_{3}^{-}$ depleted/ ${\text{NO}}_{2}^{-}$ replete conditions, to ensure confinement in PBRs. Moreover, horizontal gene transfer of chloroplast transgenes to other microbes can be avoided by a codon reassignment‐based strategy in *C*.*reinhardtii* (Young and Purton, 2016). Considering that both enzyme and biomass yield can be further enhanced by genetic tools and optimized cultivation strategies (Dall’Osto *et al*. 2019; Fields *et al*. 2018; Manuell *et al*. 2007), a microalgal‐based biofactory can produce the desired amount of the CWDE of interest in shorter time and/or irrespective from seasonal constraints and/or in countries with restrictions for the release of GMO into the environment. In the future, the HC‐PTXD mix will be tested for increasing the bio‐ethanol production by yeasts (Özçimen and İnan, *et al.,* 2015) as well as further CWDEs will be added to the HC‐PTXD mix in order to enlarge the spectrum of hydrolysable agricultural wastes by concomitantly reducing the severity of chemical pretreatments. Additional candidates will include thermostable laccases (Miyazaki 2005), hemicellulases (Benedetti *et al.,* 2019a) and pectinases (Kluskens *et al*. 2005).

## Experimental Procedures

See supporting experimental procedures.

## Declarations of interests

None.

## Author contributions

M.B., L.D. and R.B. designed the experiments; M.B. performed the experiments, analysed the data and wrote the manuscript draft. S.B., P.L., Z.G. and N.H.G. designed and performed the experiments; D.B., D.L.A., M.G‐C. and L.H‐E. contributed to the design of the experiments; N.H.G. and D.B. contributed to analysis of the data; and R.B. and L.D. supervised the research and wrote the manuscript. All authors have approved the final manuscript.

**Table 1:** Estimations on yields and production costs of different recombinant CWDEs from transplastomic *C*.*reinhardtii* and *N*.*tabacum* as reported in the most recent studies

**Enzyme**	**Enzymatic activity**	**Catalytic domain**	**Expression system**	**Enzyme yield**		**Specific activity**	**Plant production cost**	**Enzyme production cost**		**Enzyme productivity**		**Thermo stability**	**Plant alterations**	**References**
				**mg/g DW**	**U/g DW**	**U/mg**	**€ kg/DW**	**€ g^‐1^**	€ (U*10^6^)^‐1^	kg (ha*y)^‐1^	U *10^6^ (ha*y)^‐1^			
T‐EG/celB	Endoglucanase	GH12	*C*.*reinhardtii*	0.025	8.7	348	3.2^a^–3.8^b^ (T10APhi, 50 µE)	128–152	367–436	1.5	522	+++	−	This work
C‐CBH/CBM3GH5	Cellobiohydrolase	GH5		0.8	18.4	23		3.9–4.7	174–206	48	1104	++		
P‐BG/celB	β–glucosidase	GH1		0.35	33.1	95		9.1–10.8	97–114	21	1986	+++		
T‐XY/XynA	Endoxylanase	GH10		0.2	8.2	51		20–23.8	390–463	9.6	492	+++		
Cel6A	Endoglucanase	GH6		ND	ND	NA	3.2^a^‐10^b^	ND	ND	NA	NA	+		Richter *et al*. (2018)
CelK1		GH5		ND	ND	NA		ND	ND	NA	NA	TL		Faè *et al*. (2017)
Cel6A		GH6	*N*.*tabacum*	40	ND	NA	2^c^ (in field)	0.05	ND	324	NA	+		Schmidt *et al*. (2019)
CelK1		GH5		140	12	0.08*		0.014	166.7	1134	97.2	TL		Faè *et al*. (2017)
celD		GH9		100*	49300	493		0.02	0.04	810	399330	+		Verma *et al*. (2010)
EG		GH5		ND	ND	NA		ND	ND	NA	NA	++	##	Castiglia *et al*. (2016)
celB	β‐glucosidase	GH1		57*	14508	255		0.035	0.14	NA	NA	+++	#/−	
Xyn	Endoxylanase	GH10		15.5*	1633	105		0.13	1.2	125.5	13227.3	++	−	
Bgl1	β‐glucosidase	GH3		ND	444	NA		ND	4.5	NA	6798.7	TL	@	Jin *et al*. (2011)
PelB	Pectin lyase	PL1		105*	256	2.42		0.019	7.8	855	2070		−	Verma *et al*. (2010)
PelD				140*	324	2.31		0.014	6.2	1137	2627			

Annotation of glycoside hydrolase (GH) and pectin lyase (PL) domains are in accordance with the CAZy database (www.cazy.org/). CMC, *p*NPG, xylan and sodium polygalacturonate were used as substrate to determine cellulase, β‐glucosidase, xylanase and pectin lyase activity, respectively. Enzyme units (µmol min^‐1^) of HCWDEs were evaluated at 75°C. As normalization, dry weight (DW) was considered as 10% fresh weight of the plant biomass. Enzyme productivity expressed as (kg or million units) (ha*y)^‐1^ was not evaluated for plants with growth defects.*, values calculated from the available data in the original manuscript. Enzyme productivity was calculated using 8.1 and 15.3 ton DW (ha*y)^‐1^ as reference for tobacco biomass productivity where the higher value was referred to Bgl1‐expressing plants, and 60 ton DW (ha*y)^‐1^ as reference for microalgae biomass productivity. [ND: not determined, NA: not applicable, +++: hyperthermostable, ++: highly thermostable, +: thermostable, TL: thermolabile, #: stunted growth, ##: stunted growth and other developmental alterations, −: no macroscopical growth defects, @: improved biomass yield]. Cel6A is from *Thermobifida fusca*, CelK1 is from Paenibacillus sp., celD is from *Clostridium thermocellum*, EG is from *Sulfolobus solfataricus*, Xyn is from *Alicyclobacillus acidocaldarius*, Bgl1 is from *Trichoderma reesei*, and PelB and PelD are from *Erwinia carotovora*.

^a^ Tredici *et al*. (2016).

^b^ Slade and Bauen (2013).

^c^ Schmidt *et al*. (2019).

## Supporting information


**Figure S1** *C*.*reinhardtii* as biofactory of HCs.
**Figure S2** Evaluation of homoplasmic condition in four putative C‐CBH expressing transformants.
**Figure S3** Evaluation of enzymatic activity of C‐CBH from transplastomic *C*.*reinhardtii*.
**Figure S4** Immuno‐decoration analysis of cell extracts.
**Figure S5** Growth of *C*.*reinhardtii* transgenic lines using phosphite as a unique phosphorous source.
**Figure S6** Solubilization of PASC upon treatment with algal extracts.
**Figure S7** Biogas released from methanogenic bacteria culture, fed with dried WT and HC‐PTXD microalgae.
**Figure S8** Effect of HC‐PTXD mix extract on *C*.*vulgaris* growth.
**Figure S9** Degradation of pretreated corn cob flour by HC‐PTXD mix.
**Figure S10** Degradation of TT/AK‐TT corn cob flour by HC‐PTXD mix.
**Figure S11** Biomass production of *C*.*vulgaris* fed with hydrolysates from TT/AK‐TT corn cob flour and corn bran.
**Method S1** Plasmid construction.
**Method S2** *C*.*reinhardtii* transformation and selection of HC‐PTXD strains.
**Method S3** Protein extraction and enzyme activity assay.
**Method S4** Purification of HCs and determination of enzyme activity.
**Method S5** Culture conditions of *C*.*reinhardtii* strains.
**Method S6** Pretreatment of PASC with dry microalgal biomass.
**Method S7** Determination of carbohydrates in supernatants and in solid fractions.
**Method S8** Determination of biogas production from methanogenic bacteria.
**Method S9** Growth analysis of *C*.*vulgaris*.
**Method S10** Pretreatment of corn cob flour and milled bran with dry microalgal biomass.
**Method S11** Statistics.
**Table S1** Specific activity of HCWDEs towards 1% CMC, 5 mM *p*NPG, 1% xylan and 5 mM *p*NPC as determined by activity assay.
**Table S2** Specific activity of HCWDEs towards different cellulosic substrates.
