# Supplementary material for: A microalgal‐based preparation with synergistic cellulolytic and detoxifying action towards chemical‐treated lignocellulose
Source: Plant Biotechnol J. 2020 Sep 2;19(1):124–37. doi: 10.1111/pbi.13447 (PMC7769238; doi:10.1111/pbi.13447)
Supplement: Supplementary file 1 — Figure S1 C.reinhardtii as biofactory of HCs. Figure S2 Evaluation of homoplasmic condition in four putative C‐CBH expressing transformants. Figure S3 Evaluation of enzymatic activity of C‐CBH from transplastomic C.reinhardtii. Figure S4 Immuno‐decoration analysis of cell extracts. Figure S5 Growth of C.reinhardtii transgenic lines using phosphite as a unique phosphorous source. Figure S6 Solubilization of PASC upon treatment with algal extracts. Figure S7 Biogas released from methanogenic bacteria culture, fed with dried WT and HC‐PTXD microalgae. Figure S8 Effect of HC‐PTXD mix extract on C.vulgaris growth. Figure S9 Degradation of pretreated corn cob flour by HC‐PTXD mix. Figure S10 Degradation of TT/AK‐TT corn cob flour by HC‐PTXD mix. Figure S11 Biomass production of C.vulgaris fed with hydrolysates from TT/AK‐TT corn cob flour and corn bran. Method S1 Plasmid construction. Method S2 C.reinhardtii transformation and selection of HC‐PTXD strains. Method S3 Protein extraction and enzyme activity assay. Method S4 Purification of HCs and determination of enzyme activity. Method S5 Culture conditions of C.reinhardtii strains. Method S6 Pretreatment of PASC with dry microalgal biomass. Method S7 Determination of carbohydrates in supernatants and in solid fractions. Method S8 Determination of biogas production from methanogenic bacteria. Method S9 Growth analysis of C.vulgaris. Method S10 Pretreatment of corn cob flour and milled bran with dry microalgal biomass. Method S11 Statistics. Table S1 Specific activity of HCWDEs towards 1% CMC, 5 mM pNPG, 1% xylan and 5 mM pNPC as determined by activity assay. Table S2 Specific activity of HCWDEs towards different cellulosic substrates. [file PBI-19-124-s001.docx]

**Supporting information**

**Figure S1**

**
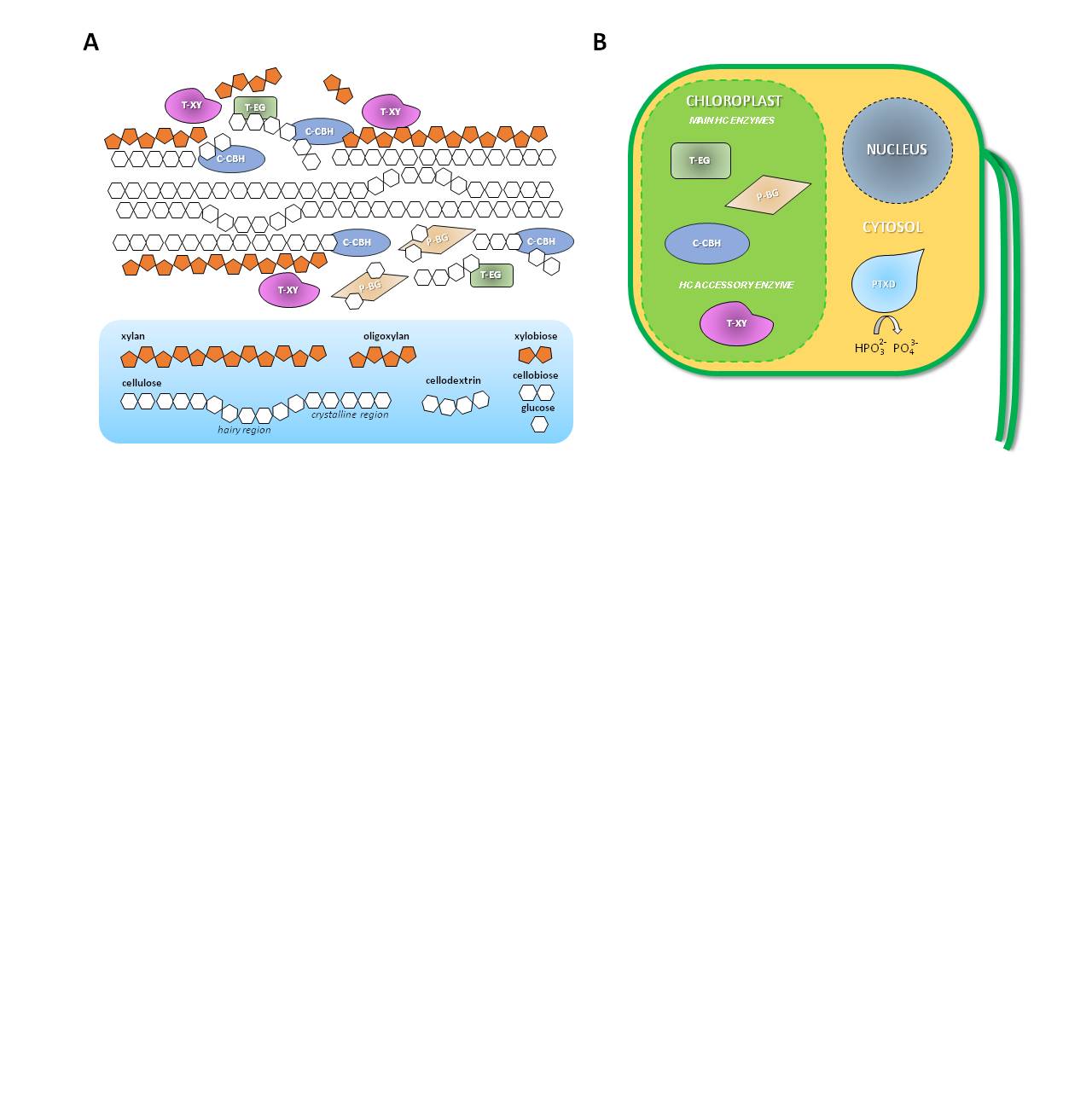
**

**Figure S1. *C. reinhardtii* as biofactory of HCs.** (A) Enzymatic deconstruction of xylan-cellulose assembly as expected by the selected hyperthermophilic CWDEs. (B) Schematic representation of the transgenic *C. reinhardtii* cell. The enzymes constituting the cellulolytic machinery are reported*.* Each strain co-expresses PTXD and one HC-enzyme. [T-EG=Endoglucanase from *T. neapolitana*, C-CBH=Cellobiohydrolase from *C. saccharolyticus*, P-BG=beta-glucosidase from *P. furiosus*, T-XY=Xylanase from *T. neapolitana,* PTXD=Phosphite Dehydrogenase from *P. stutzeri* WM88].

**Figure S2**


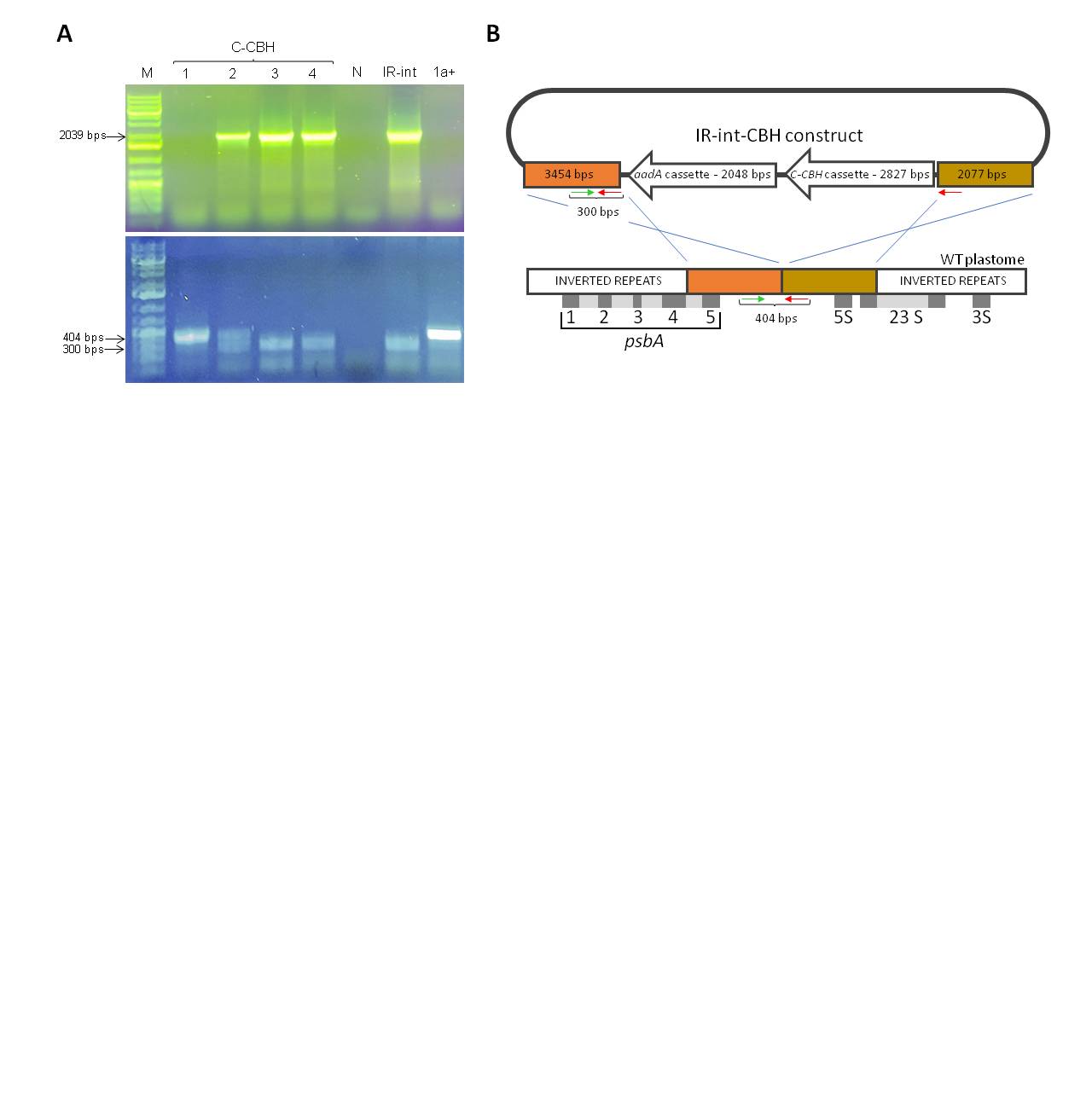


**Figure S2. Evaluation of homoplasmic condition in four putative C-CBH expressing transformants.** (A) (*upper panel*) Presence of the transgene (2039 bps) in four selected transformants (1-4) as determined by PCR. Wild type gDNA (1a+) and IR-int-CBH construct (IR-int) were used as negative and positive control, respectively. (*lower panel*) Amplicons of 404 bps and of 300 bps from *C. reinhardtii* plastome showed the presence of endogenous inverted repeats (IR) or integrated plasmid, respectively. Wild type gDNA (1a+) and IR-int-CBH construct (IR-int) were used as negative and positive control of homoplasmic condition, respectively. Analysis of four C-CBH selected transformants (1-4) is presented as representative result. Molecular weight marker (M) and negative control of the PCR reaction (N) are also reported. (B) Map of the insertion site of IR-int-CBH construct in the inverted repeats of wild type plastome by homologous recombination. Binding sites of forward and reverse primers used for the screening of homoplasmic transformants are reported as green and red arrows, respectively. The homoplasmic condition of IR-int transformants was confirmed by the absence of specific amplification product (404 bps). The psbA and the ribosomal RNA genes (5S, 23S, 3S) are also shown as boxes with exons in dark grey and introns in light grey. The scheme is readapted from Michelet et al., 2011.

**Figure S3**

**
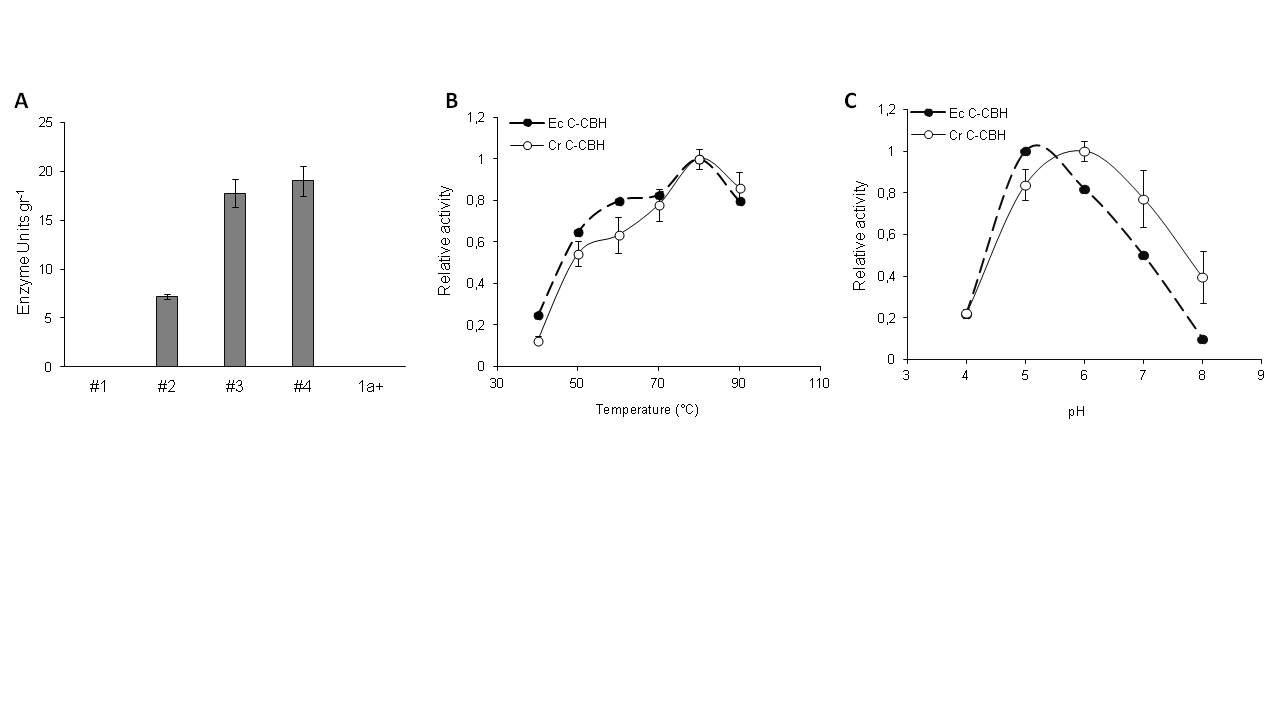
**

**Figure S3.** **Evaluation of enzymatic activity of C-CBH from transplastomic *C. reinhardtii*. (A)** Quantification of C-CBH activity in protein extracts from the *C. reinhardtii* transformant lines shown in Supplementary Figure S2. Activity was evaluated towards 1% CMC and expressed as Units (µmol min^-1^) per g DW of microalga. (B, C) Effect of temperature (B) and pH (C) on the activity of C-CBH, purified from either the homoplasmic transformant #3 (Cr C-CBH) or transgenic *E. coli* (Ec C-CBH); activity values of Ec C-CBH, here used as reference, have been extrapolated from (Park et al. 2011); curves were normalized to 1 at the maximum. Protein extracts were obtained by treating the dry biomass with non-ionic detergent and heat (10 mM citrate pH 5.5, 0.3% Tween20, incubated for 1 hour at 70°C). Data are expressed as mean ± SD, n = 3. 1a+, wild type strain.

**Figure S4**


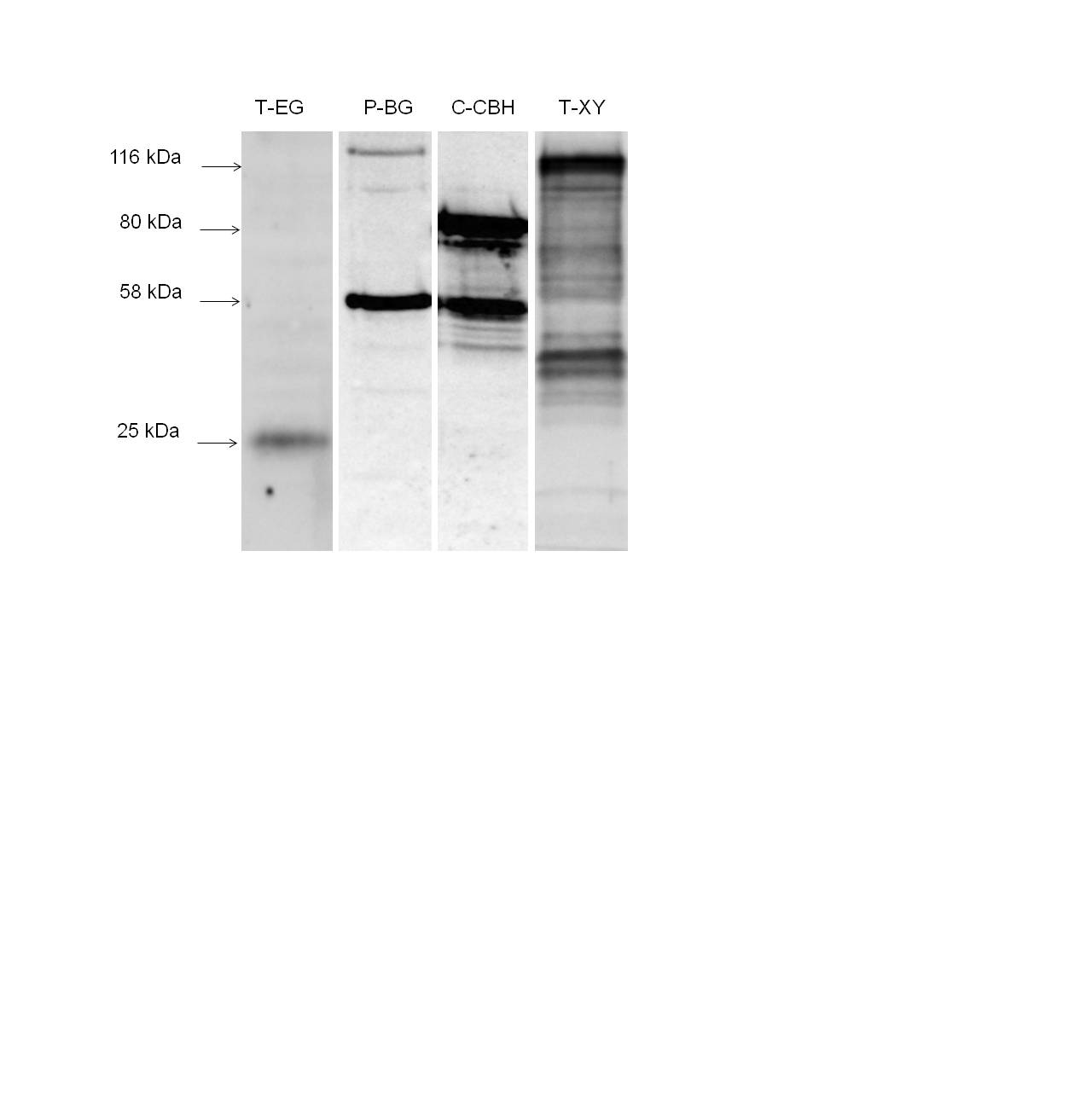


**Figure S4. Immuno-decoration analysis of cell extracts.** Western blot was performed on cell extracts obtained by non-ionic detergent + heat treatment (10 mM citrate pH 5.5, 0.3% Tween20, incubated for 1 hour at 70°C) of transgenic strains. Total proteins (60 µg/lane) from 7-days old cultures were assayed using α-HA primary antibody. Molecular weight of the bands detected are indicated on the left. Abbreviations: T-EG, endoglucanase B from *Thermotoga neapolitana,* 27 kDa; P-BG, β-glucosidase from *Pyrococcus furiosus*, 58 kDa; C-CBH, Cellobiohydrolase from *Caldicellulosiruptor saccharolyticus*, 80 kDa; T-XY, Endoxylanase from *Thermotoga neapolitana,* 116 kDa.

**Figure S5**

**
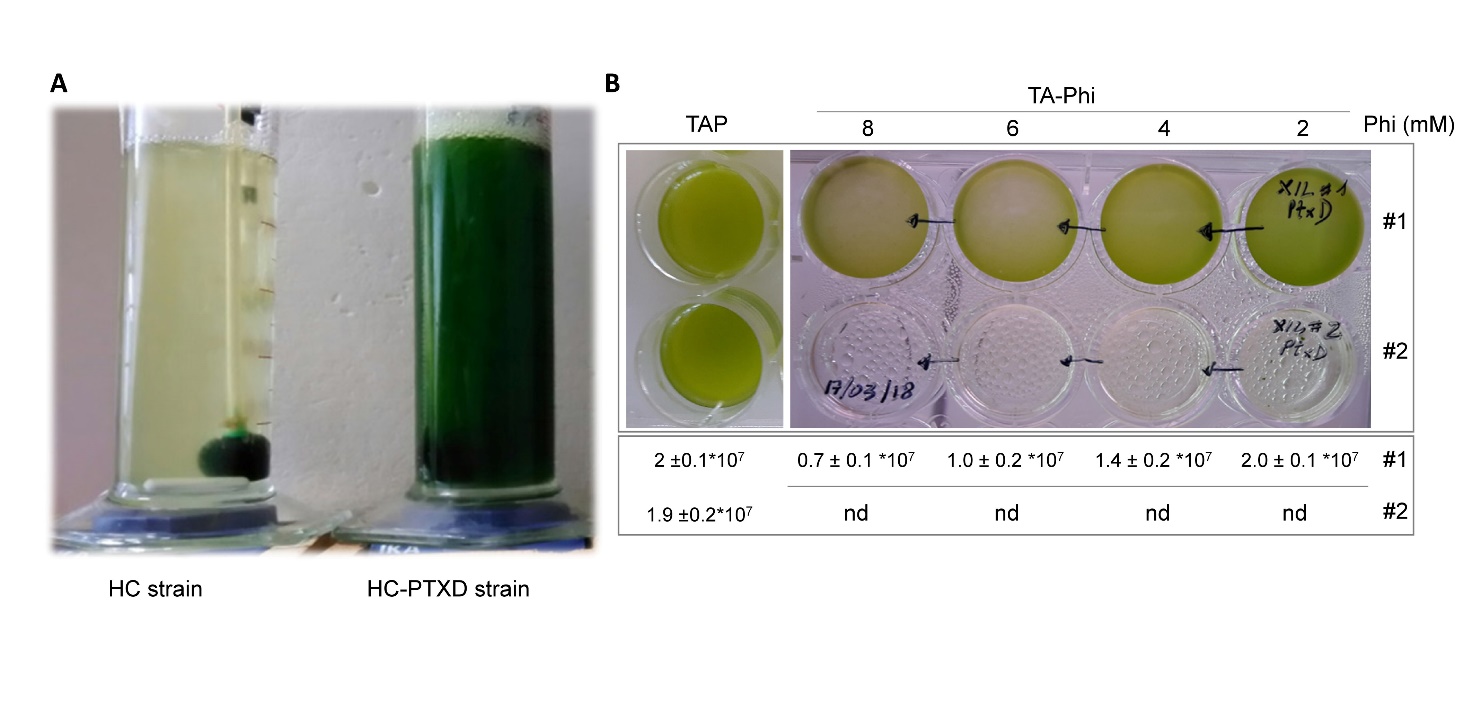
Figure S5. Growth of *C. reinhardtii* transgenic lines using phosphite as a unique phosphorous source.** (A) HC strain (left) and HC-PTXD strain (right) were grown for 7 days at 200 µmol photons m^-2^ s^-1^, 24°C in a phosphate-depleted/phosphite-repleted medium under mixotrophic conditions (TA-Phi medium). (B) (*upper panel*) 7-days old cultures of two zeocin-resistant HC-PTXD transformants (#1, #2), grown in TA medium supplemented with increasing amounts of Phi (2-8 mM) as unique phosphorous source. T-XY-PTXD #1 is reported as representative HC-PTXD line showing robust growth in Phi-supplemented media whereas T-XY-PTXD #2 is reported as zeocin-resistant transformant unable to grow in Phi-supplemented media. Cultures in TAP medium are reported as positive control of growth. (*lower panel*) Cell densities, expressed as cells mL^-1^, of the cultures shown in the upper panel. Starting inoculum was 2.5 · 10^5^ cells mL^-1^. Growth was performed at 50 µmol photons m^-2^ s^-1^, 24°C with orbital shaking at 150 rpm. Data are expressed as mean ± SD, n = 2. nd, not detected.

**Figure S6**

**
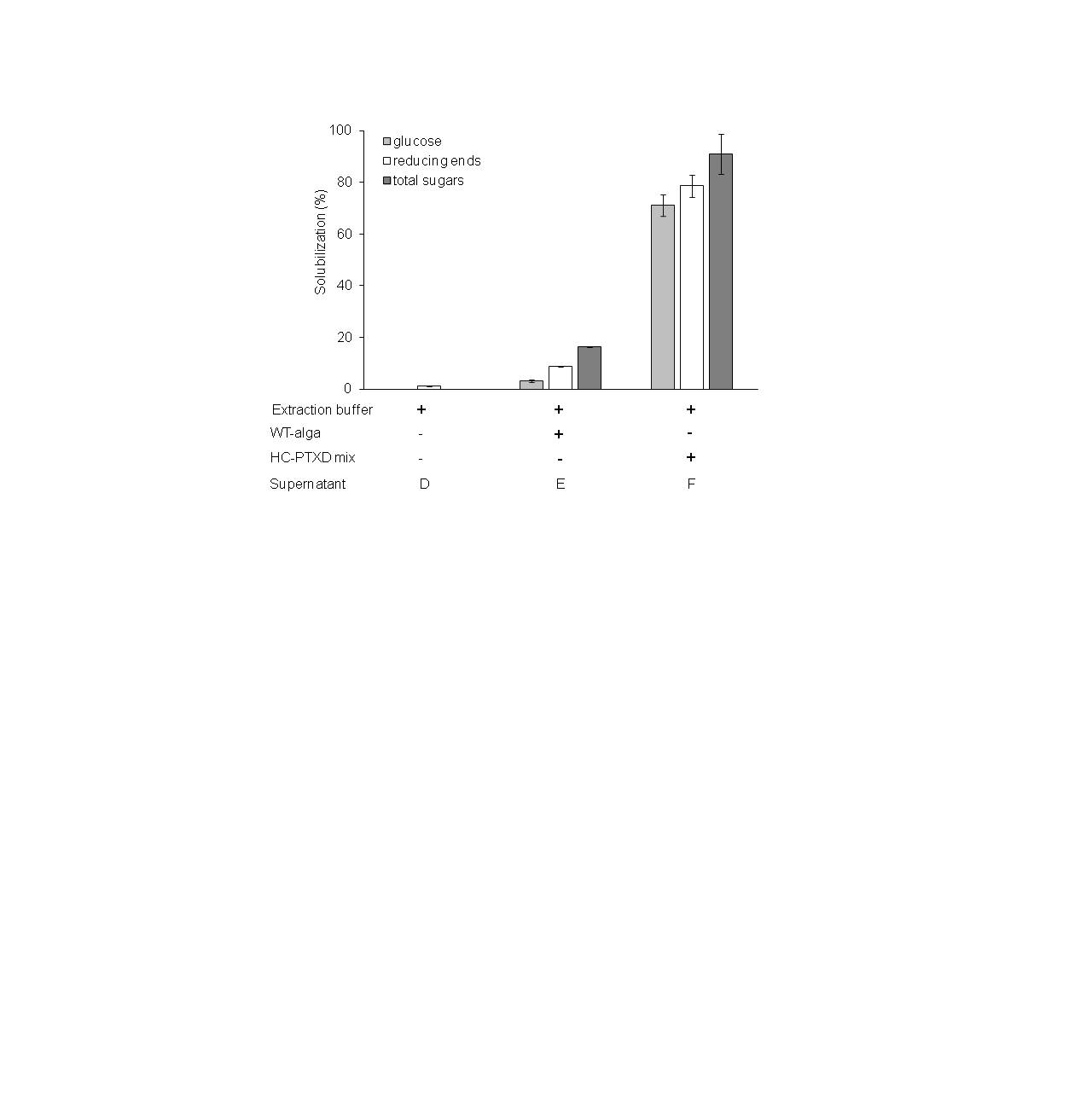
**

**Figure S6. Solubilization of PASC upon treatment with algal extracts.** Solubilization of PASC (0.3% w/v) into glucose (light gray bar), reducing ends (white bar) and sugars (dark gray bar) was determined after 1-day incubation with either the only extraction buffer (supernatant D), with the extracts from wild type microalga (supernatant E) or from HC-PTXD mix (supernatant F). See methods for details. Data are expressed as mean ± SD, n = 3.

**Figure S7**

**
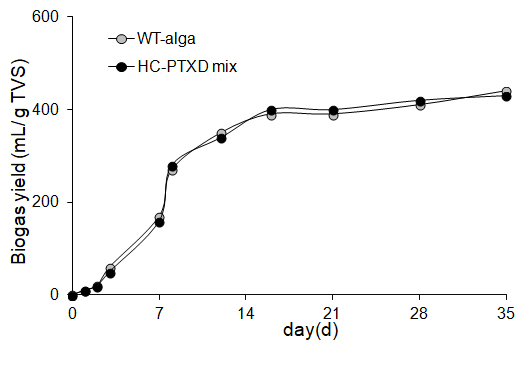
**

**Figure S7. Biogas released from methanogenic bacteria culture, fed with dried WT and HC-PTXD microalgae.** 0.6 g DW of algal powder from *C. reinhardtii* wild type or HC-PTXD mixture were supplemented to 100 mL of methanogenic bacteria suspension from digested sludge. Quantification of bio-methane produced and analysis of total volatile solids (TVS) were performed as reported in Methods section. Values of biogas yield were already subtracted to those of the control reaction (production of biogas without algal powder supplementation).

**Figure S8**

**
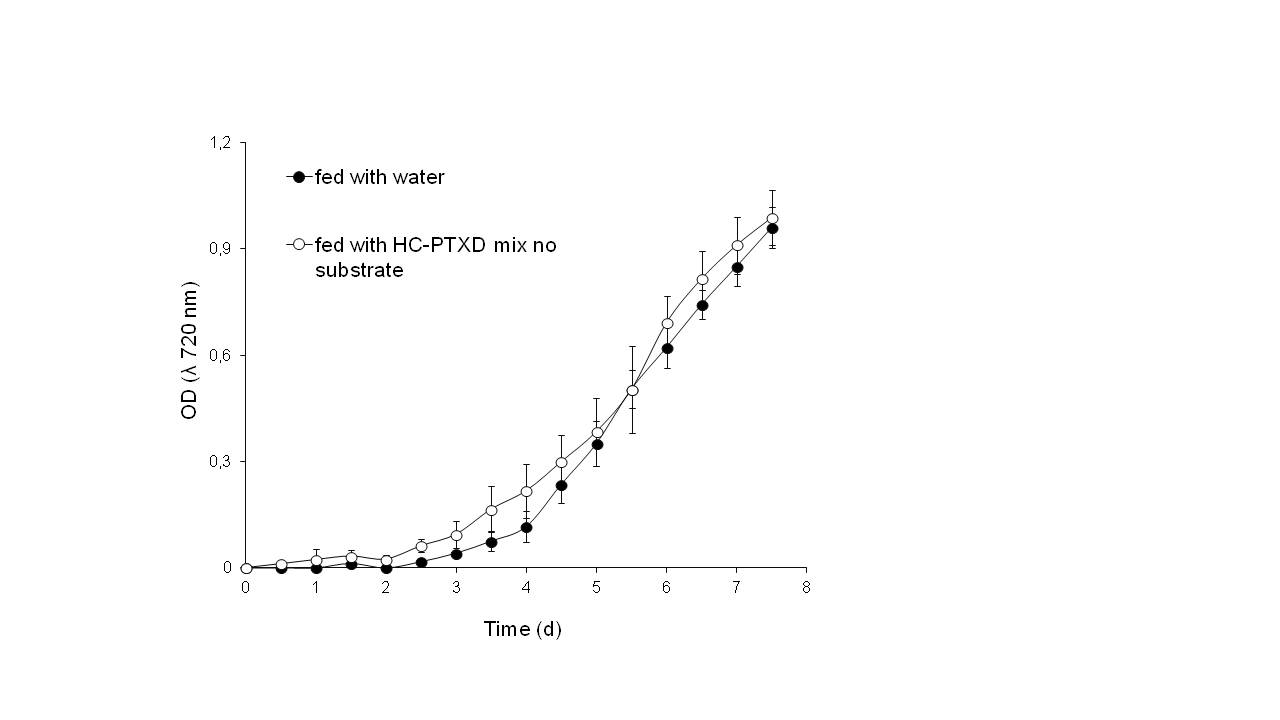
**

**Figure S8. Effect of HC-PTXD mix extract on *C. vulgaris* growth.** Growth curves of *C. vulgaris* were obtained by measuring the optical density (OD) at 720 nm. Cultures were fed with either water or HC-PTXD mix extract (HC-PTXD mix no substrate). 2.5∙10^5^ cells mL^-1^ were used as starting inoculum. Data are reported as mean ± SD, n = 3. No significant differences on growth were measured between the two conditions.

**Figure S9**

**
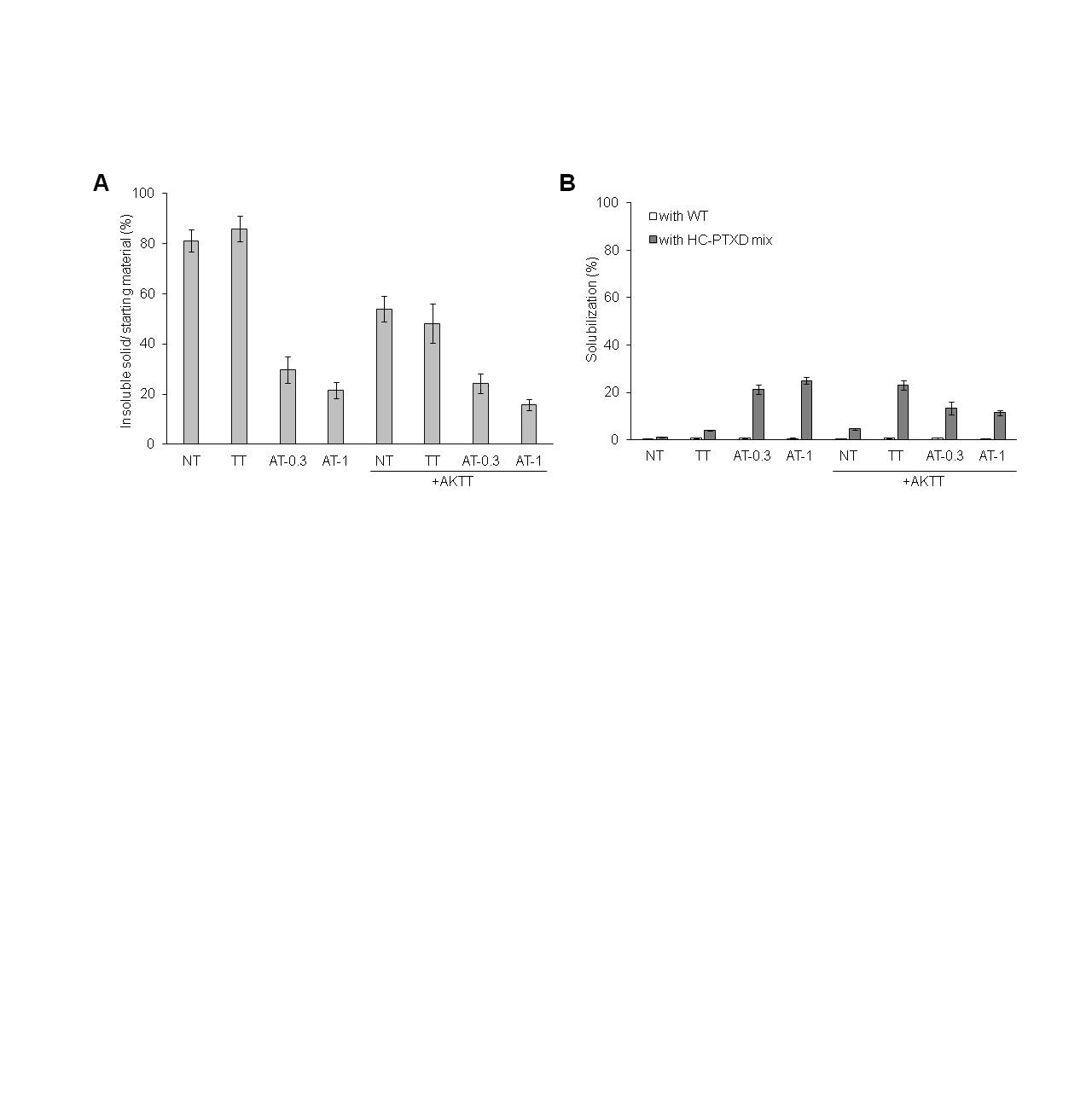
**

**Figure S9. Degradation of pretreated corn cob flour by HC-PTXD mix.** (A) Weight percentage of insoluble solid fraction recovered from corn cob flour upon different physico-chemical treatments. (B) Solubilization of insoluble solid fractions from differently pretreated corn cob flour, upon 1-day incubation (0.8% w/v) with extracts from either wild type microalga (white bar) or HC-PTXD mix (gray bar). Abbreviations: NT, No Treatment; TT, Thermic Treatment; ATT-0.3, Acid (H_2_SO_4_ 0.3% w/v)-Thermic Treatment; ATT-1, Acid (H_2_SO_4_ 1% w/v)-Thermic Treatment; +AKTT; plus Alkaline (NaOH 4% w/v) -Thermic Treatment. See methods for details. Data are expressed as mean ± SD, n = 3.

**Figure S10**

**
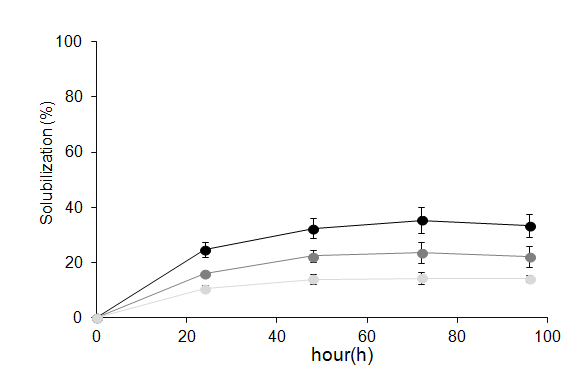
**

**Figure S10. Degradation of TT/AK-TT corn cob flour by HC-PTXD mix.** Time-course analysis of solubilization of TT/AK-TT-treated corn cob flour (0.8% w/v) into sugars (black line), reducing ends (dark gray line) and glucose (light gray line) by the extract from HC-PTXD mix. Solubilization was carried out at 75°C, see methods for further details. Data are expressed as mean ± SD, n = 3.

**Figure S11**

**
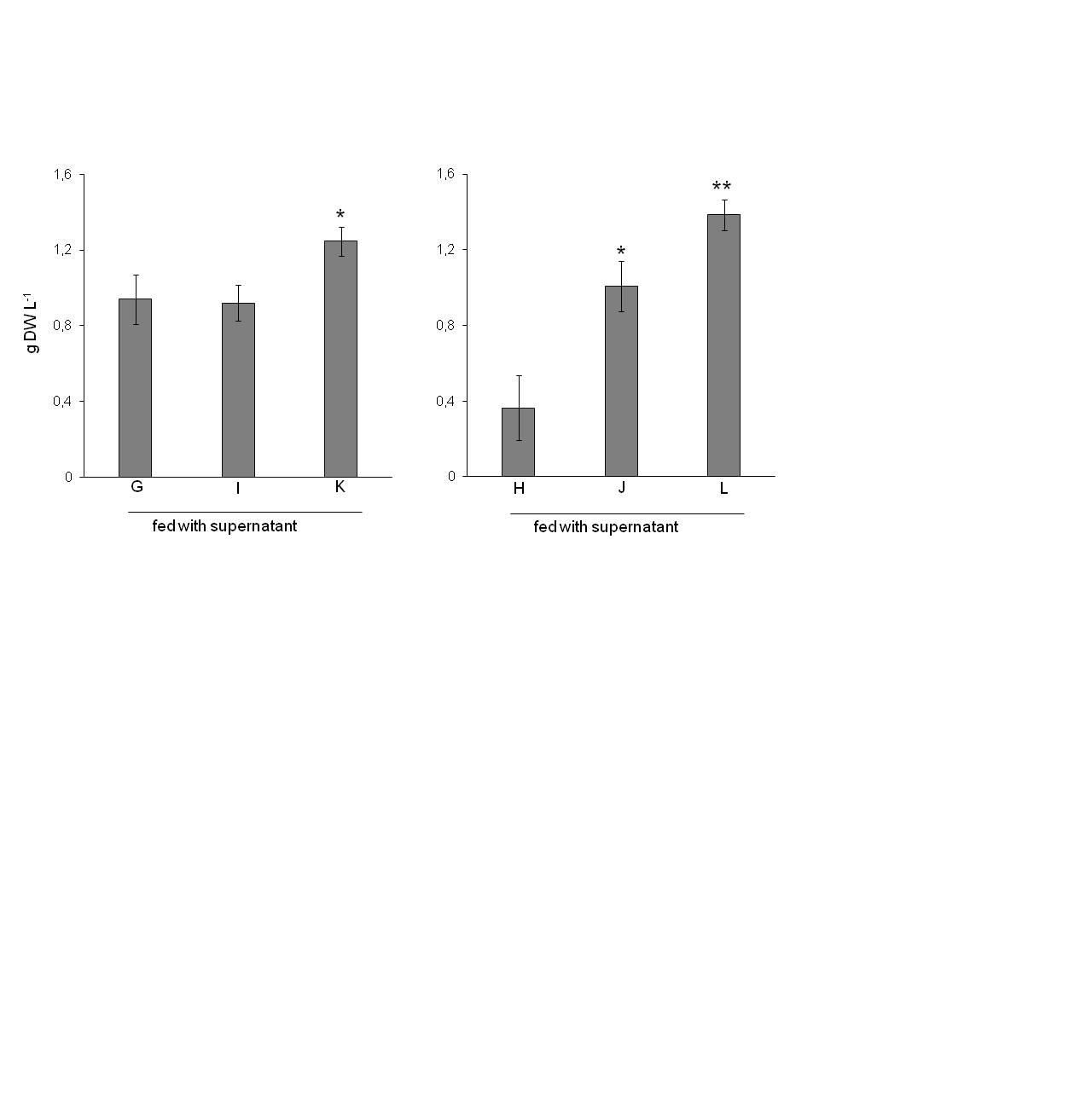
**

**Figure S11. Biomass production of *C. vulgaris* fed with hydrolysates from TT/AK-TT corn cob flour and corn bran.** Biomass determination from the same *C. vulgaris* cultures described in Figure 5C (*left panel*) and Figure 5D (*right panel*), after 12 days of growth. Solubilization of TT/AK-TT corn cob flour and bran (0.8% w/v) was performed by incubating the substrate for 2-days with either the extraction buffer only (supernatant G, H), the extracts from wild type microalga (supernatant I, J) or the extracts from HC-PTXD mix (supernatant K, L). Data are expressed as mean ± SD, n = 3. In each panel, significantly different values (Student’s *t* test, * P < 0.05, ** P < 0.01) against controls fed with either supernatant G or H, are marked.

.

**SUPPORTING EXPERIMENTAL PROCEDURES**

**Method S1 Plasmid construction**

Four different HCWDEs were selected as part of our system. CelB (T-EG) from *T. neapolitana* (UniprotKB: P96492, aa 18-274), the CBM3GH5 portion of CelB (C-CBH) from *C. saccharolyticus* (UniprotKB: P10474, aa 380-1039), β-glucosidase (P-BG) from *P. furiosus* (UniprotKB: Q51723, aa 1-472) and Xylanase XynA (T-XY) from *T. neapolitana* (UniprotKB: Q60042, aa 30-1055) were reverse-translated into codon-optimized sequences by using the software OPTIMIZER (http://genomes.urv.es/OPTIMIZER/) (Puigbo et al. 2007) according to the codon usage of *C. reinhardtii* chloroplast. Putative signal peptides of selected HCWDEs were identified by using the Signal IP 4.1 Server (http://www.cbs.dtu.dk/services/SignalP/) (Petersen et al. 2011) and excluded from such manipulation. The sequence encoding for the HA-epitope (YPYDVPDYA) was added to the 3' of each CWDE sequence. All these genes were synthesized by GeneArt (Life Technologies). Synthetic genes were separately cloned downstream of the *psaA* promoter into the AtpB-int vector (Faè et al. 2017). Subsequently, the expression cassette was excised from AtpB-int using the restriction enzymes ClaI and SmaI and cloned in the IR-int vector (Michelet et al. 2011, Faè et al. 2017). *E. coli* strain XL10gold (Agilent Technologies) was transformed and used for plasmid propagation.

**Method S2 *C. reinhardtii* transformation and selection of HC-PTXD strains.**

The green microalga *C. reinhardtii* wild type 1a (mt +) was obtained from the Chlamydomonas Resource Center (University of Minnesota). This strain was cultured at 25°C in liquid TAP (Tris-HCl 2.4 g L^-1^, Acetic acid 1 g L^-1^, 1 mM K_2_HPO_4_, see Kropat et al. 2011), under illumination of 50 µmol photons m^-2^ s^-1^ white light, 16/8 hrs light/dark photoperiod and orbital shaking (150 rpm). The IR-int vector used in this study contains a 7-Kbps region from the *C. reinhardtii* plastome (i.e., the “inverted repeats”, abbreviated “IR”) flanking the expression cassette of the transgene. This IR region is required for homologous recombination in the plastome of *C. reinhardtii* upon transformation. For a detailed description of the IR-int vector as well as of the genetic rearrangements in the *C. reinhardtii* plastome, see (Michelet et al. 2011; Faè et al. 2017). Plastid transformation and the selection of homoplasmic lines were carried out as in (Faè et al. 2017). HC-expressing strains were transformed by electroporation using the construct pChlamy4-PTXD from (Loera-Quezada et al. 2016). Briefly, cells of each HC-expressing strain from the exponential phase were collected by centrifugation and washed with MAX Efficiency™ Transformation Reagent for Algae (ThermoFisher Scientific), then resuspended in the same reagent at a final concentration of 2*10^8^ cells mL^-1^. A total of 1.5 μg of ScaI‐linearized construct was added to the cells for each transformation, and 250 μL of the transformation mixtures were transferred into a 4‐mm electroporation cuvette and incubated at 4°C for 5 min. The electroporation parameters were set as follows: 500 V, 50 μF and 800 Ω. After electroporation, cells were mixed with 5 mL of TAP‐40 mM sucrose solution and incubated for 5 h to let them recover from electroporation. Transformants were selected on TAP agar plates containing 20 μg mL^-1^ zeocin as selective agent. Subsequently, the identification of the PTXD-expressing transformants was carried out by monitoring the growth of the zeocin-resistant clones in liquid TA-Phi medium (Tris-HCl 2.4 g L^-1^, Acetic acid 1 g L^-1^, 1 mM potassium phosphite monobasic), i.e., a modified version of TAP medium in which the phosphate (KH_2_PO_4_) was replaced by phosphite (KH_2_PO_3_) as sole phosphorous source. KH_2_PO_3_ was purchased from Wanjie Int., China (CAS No. 13977-65-6). Transformants capable of growing in such condition were inoculated in liquid TA-Phi medium supplemented with increased concentration of KH_2_PO_3_. Only the lines showing a robust growth up to 4 mM KH_2_PO_3_ were selected as the HC-PTXD strain. Growth was also followed by measuring cell density (Countess II FL CellCounter, ThermoFisher). *C. reinhardtii* wild type strain was found unable to grow in KH_2_PO_3_-supplemented media, in agreement with (Loera-Quezada et al. 2016).

**Method S3 Protein extraction and enzyme activity assay**

Cells were collected by centrifugation, freeze-dried, then resulting powder was stored at -80°C. Protein extraction was performed by using different procedures. In all the methods tested, dry biomass was resuspended according to the ratio [1 mL extraction buffer:6 mg microalgal powder]. Extraction in non-denaturing condition was performed in 10 mM citrate buffer pH 5.5 and 0.3% Tween20, then the suspension was incubated under gentle shaking for 1 h at 70°C. Extraction by mechanical destruction was carried out by treating the former suspension for 30 min in ultrasonic bath with glass beads 425-600 µm (Sigma-Aldrich) at RT. Extraction in denaturing condition was performed in 20 mM Tris-HCl buffer pH 7.0, 2% SDS and 10 mM EDTA and the suspension was then incubated under gentle shaking for 30 min at RT. Extraction by heat was performed in water and the suspension was then incubated under gentle shaking for 1 h at 70°C. At the end of each procedure, the sample was centrifuged (14,000g x 10 min) and the supernatant was used for downstream applications. Proteins extracted from 120 µg DW of biomass were separated in 10% of Laemmli gel then either visualized by Coomassie Blue staining or transferred to nitrocellulose membrane (Towbin et al. 1979) and immuno-detected with a monoclonal AbHA (HA7 clone, Sigma-Aldrich). Enzymatic activity was assayed by incubating the algal extracts (10% v/v, 100 µl total volume) or the partially purified enzymes (10-50 ng) in 50 mM Na-Acetate buffer pH 5.5, at either 75°C or as indicated in (Kengen et al. 1993; Zverlov et al. 1996; Bok et al. 1998; Park et al. 2011) by using the following substrates: 1% w/v carboxy-methylcellulose (CMC) and 5 mM *p*-nitrophenyl-β-cellobioside (*p*NPC) to determine cellulase activity, 5 mM *p*-nitrophenyl-β-glucopyranoside (*p*NPG) to determine β-glucosidase activity, 1% w/v xylan from beechwood to determine xylanase activity. All substrates were purchased from Sigma-Aldrich. CMC was also used to determine the effect of pH and temperature on the activity of C-CBH, in accordance with (Park et al. 2011). Enzyme activity was expressed as Enzyme Units (µmol of reducing sugar equivalents released per min, or µmol of *p*-nitrophenol released per min) per g DW of microalga. Determination of µmol reducing ends released upon hydrolysis was performed according to (Lever 1972) using different amounts of glucose as calibration curve. Determination of µmol *p*-nitrophenol released upon hydrolysis was determined using different amounts of *p*-nitrophenol as calibration curve. Enzyme Units were expressed as mean of the values determined at two different time-points.

**Method S4 Purification of HCs and determination of enzyme activity**

100 mg DW of *C. reinhardtii* powder were resuspended in non-denaturing buffer (10 mM Tris-HCl pH 7.5, 0.3% Tween20) in the ratio [1 mL extraction buffer:6 mg DW microalgal powder], then incubated for 1 hour at 70°C, centrifuged (14,000 g x 10 min) and the supernatant was loaded on Q-sepharose column (Amersham) equilibrated with 20 mM Tris-HCl, pH 7.5. Elution was performed by using a step-wise gradient of NaCl and the eluted fractions were then tested by the appropriate enzymatic activity assay. Fractions that displayed the highest activity were analyzed by SDS-PAGE. Quantification of the amount of each HCs was performed in Coomassie-stained gel by comparing the intensity of specific bands (identified by immuno-blot analysis) with a standard curve of BSA (Quantity-One software, Biorad). Determination of specific activity allowed to evaluate the expression level of each enzyme from the corresponding HC-expressing strain. Control assays included substrate without algal extract or algal extract without substrate.

**Method S5 Culture conditions of *C. reinhardtii* strains**

*C. reinhardtii* was grown at 50 µmol photons m^-2^ s^-1^ white light, 25°C, in flasks or 6-well culture plates maintained in an orbital shaker (150 rpm), or photobioreactor system (MC-1000, Photon System Instruments) with air bubbling supplementation. Biomass production assays were performed in home-built 1-L indoor photobioreactors (Cazzaniga et al. 2014). Growth assays of HC-PTXD strains were performed in different liquid media including HS medium (Sueoka, 1960), TAP medium (Kropat et al. 2011), TA-Phi medium and T10A-Phi medium, with the latter a modified version of TA-Phi medium containing 0.24 g L^-1^ Tris, corresponding to 1/10 of the concentration in conventional TAP/TA-Phi media. Growth in phosphite-supplemented media was performed in unsterilized systems.

**Method S6 Pretreatment of PASC with dry microalgal biomass**

PASC (Phosphoric Acid Swollen Cellulose), prepared as described in (Cannella et al. 2016), was characterized by a sugar content greater than 77% (w/w). 0.6 g of dry HC-PTXD mixture were resuspended in 100 mL of non-denaturing extraction buffer, incubated at 70°C for 1 h, then the supernatant was collected upon centrifugation (14,000g x 10 min). PASC was added to the supernatant (0.3 or 0.6%, w/v) and incubated at 75°C for 24 h. In order to assess the durability of the enzymes, the reaction mix was supplemented with fresh PASC (0.6% w/v) every 24 hours, and the procedure was repeated for four cycles. Control assays included substrate without algal extract or algal extract without substrate.

Before sugar analysis, the reaction mixtures were centrifuged (4,000g, 5 min) and the supernatants, here termed as hydrolysates, were filter-sterilized and used for downstream applications. Values of solubilization (%) expressed the weight percentage of either glucose, reducing ends equivalents or total sugars, released from PASC. All the supernatants from enzymatic reactions were named with letters in alphabetical order (A-F) according to their first occurrence in the text.

**Method S7 Determination of carbohydrates in supernatants and in solid fractions**

Determination of reducing ends released upon hydrolysis was performed according to (Lever 1972) using difference amounts of glucose as calibration curve. Glucose content was quantified by a glucose-oxidase/peroxidase assay (GOPOD assay kit, Megazyme). Total sugars were estimated using the phenol-sulfuric acid assay (Dubois et al. 1956). In solid fractions, total carbohydrates were determined upon acid hydrolysis according to (http://www.nrel.gov/biomass/analytical_procedures.html): samples were first hydrolyzed in 72% (v/v) H_2_SO_4_ at 30 °C for 1 h and then in 4% (v/v) H_2_SO_4_ at 120 °C for 1 h.

**Method S8 Determination of biogas production from methanogenic bacteria**

Bio-methane production assays were carried out by supplementing 25 mL of the filter-sterilized supernatant from treated PASC to 100 mL of methanogenic bacteria suspension from digested sludge. Composition of the digested sludge was the same as that used in (Herrero-Garcia et al. 2019). Experiments of methanogenesis were carried out using glass bottles of half liter, operating without pH control in batch mode. Quantification of bio-methane produced, analysis of total volatile solids (TVS) and determination of Chemical Oxygen Demand (COD) were performed as reported in (Herrero-Garcia et al. 2019).

**Method S9 Growth analysis of *C. vulgaris***

*C. vulgaris* growth tests were performed in a multi-cultivator system (Photon System Instruments) under air bubbling supplementation, at 25°C in BG-11 liquid medium (Kuhl and Lorenzen 1964). A 70-mL culture of *C. vulgaris* wild-type strain 211-11p (Culture Collection of Algae, Göttingen University, Germany) was supplemented with 10 mL of filter-sterilized supernatant from either PASC or TT-AKTT corn samples treated with dry microalgal biomass. The parameters determined to monitor cell growth were cell number and biomass DW.

**Method S10** **Pretreatment of corn cob flour and milled bran with dry microalgal biomass**

Pretreatments of corn cob flour were carried out according to (Chen et al. 2013; Zheng et al. 2014) with the following modifications. Corn biomass was suspended in aqueous solution (7% w/v) and treated at 4 different conditions: (1) “No treatment” (NT), corn cob flour was suspended in water; (2) “Thermic Treatment” (TT), corn cob flour was suspended in water and autoclaved for 1h; (3,4) “Acid-Thermic Treatment” (AT), corn cob flour was suspended in either 0.3% (AT-0.3) or 1% (AT-1) H_2_SO_4_ and autoclaved for 1 h, then neutralization was performed by adjusting pH to 5.5; loading of H_2_SO_4_ was [0.04 g H_2_SO_4_: 1 g corn cob flour] and [0.14 g H_2_SO_4_: 1 g corn cob flour] for AT-0.3 and AT-1, respectively. Upon treatment and centrifugation (14000g x 10 min), the pellets collected (referred to as insoluble solids) were dried and used either (i) as substrate for enzymatic assay, or (ii) for alkaline-thermic treatment (AKTT). In the latter case, insoluble solids were treated for 2h at 65°C with 4% NaOH. After incubation, samples were centrifuged, supernatants discarded and the insoluble solids were washed with water and freeze-dried. Insoluble solids from the different reactions were subjected to enzymatic treatment at 75°C, using extracts from either wild-type or HC-PTXD dry microalgal biomass (see previous paragraph) and then sugar content was determined. The two-step procedure constituted by TT and AK-TT was selected for the pretreatment of both corn cob flour and milled corn bran (referred to as TT-AKTT cob and TT-AKTT bran). TT-AKTT cob and TT-AKTT bran samples were characterized by a sugar content greater than 90%. Enzymatic treatment of TT/AK-TT samples was performed as follows: 0.6 g DW from either wild-type or HC-PTXD mix was dissolved in 100 mL of non-denaturing extraction buffer and incubated at 70°C for 1 h. The supernatant was then collected upon centrifugation (14,000g x 10 min). TT-AKTT samples were added to each supernatant (0.8% w/v), incubated at 75°C for 48h and then centrifuged (4,000g x 5 min). Before proceeding with sugar determination, the reaction mixtures were centrifuged (4,000g x 5 min) and the supernatants were filter-sterilized and used for downstream applications. Control assays included substrate without algal extract or algal extract without substrate. Values of solubilization (%) expressed the weight percentage of either glucose, reducing ends equivalents or total sugars, released from TT-AKTT materials. All the supernatants from enzymatic reactions were named with letters in alphabetical order (G-L) according to their first occurrence in the text.

**Method S11 Statistics**

Significance analysis was performed in GraphPad Prism software, using either Student’s *t* test, F-test or One-way analysis of variance (ANOVA) and means separated with Tukey’s post-test at a significant level of P < 0.05 (see the figure legends for details). Error bars represent the standard deviation.

**Table S1. Specific activity of HCWDEs towards 1% CMC, 5 mM *p*NPG, 1% xylan and 5 mM *p*NPC as determined by activity assay.** Enzyme activity, expressed as Units (µmol min^-1^) per mg of enzyme, was evaluated at 75°C. Data are expressed as mean ± SD, n = 3.

**Table S2. Specific activity towards different cellulosic substrates.** Specific activities of HC-PTXD mix towards 1% CMC (w/v), 0.6% PASC (w/v) and 2.5% Avicel (w/v). Enzyme Units correspond to µmol reducing ends min^-1^ produced per g DW of HC-PTXD mix. Data are expressed as mean ± SD, n = 3.

**Reference List**

Bok JD, Yernool DA, Eveleigh DE (1998) Purification, characterization, and molecular analysis of thermostable cellulases CelA and CelB from *Thermotoga neapolitana*. *Appl Environ Microbiol* **64**: 4774–4781.

Cannella D, Möllers KB, Frigaard, NU, Jensen, PE, Bjerrum MJ, Johansen KS, Felby C (2016) Light-driven oxidation of polysaccharides by photosynthetic pigments and a metalloenzyme. *Nat Commun* **7**: 11134.

Cazzaniga S, Dall’Osto L, Szaub J, Scibilia L, Ballottari M, Purton S, Bassi R. (2014) Domestication of the green alga *Chlorella sorokiniana*: reduction of antenna size improves light-use efficiency in a photobioreactor. *Biotechnol Biofuels.* **7**: 157.

Chen Y, Stevens MA, Zhu Y, Holmes J, Xu H (2013) Understanding of alkaline pretreatment parameters for corn stover enzymatic saccharification. *Biotechnol Biofuels* **6**: 8.

Dubois M, Gilles KA, Hamilton JK, Rebers PA, Smith F (1956) Colorimetric Method for Determination of Sugars and Related Substances. *Anal Chem* **28**: 350–356.

Faè M, Accossato S, Cella R, Fontana F, Goldschmidt-Clermont M, Leelavathi S, Reddy VS, Longoni P. (2017) Comparison of transplastomic *Chlamydomonas reinhardtii* and *Nicotiana tabacum* expression system for the production of a bacterial endoglucanase. *Appl Microbiol Biotechnol* **101**: 4085–4092.

Herrero-Garcia N, Mattioli A, Gil A, Frison N, Battista F, Bolzonella D (2019) Evaluation of the methane potential of different agricultural and food processing substrates for improved biogas production in rural areas. *Renewable and Sustainable Energy Reviews* **112**: 1-10.

Kengen SWM, Luesink EJ, Stams AJM, Zenhder, AJB. (1993) Purification and characterization of an extremely thermostable beta-glucosidase from the hyperthermophilic archaeon *Pyrococcus furiosus*. *Eur J Biochem* **213**: 305–312.

Kropat J, Hong-Hermesdorf A, Casero D, Ent P, Castruita M, Pellegrini M, Merchant SS, Malasarn D (2011) A revised mineral nutrient supplement increases biomass and growth rate in *Chlamydomonas reinhardtii.* *Plant J* **66**: 770-80.

Lever M (1972) A new reaction for colorimetric determination of carbohydrates. *Anal Biochem* **47**: 273–279

Loera-Quezada MM, Leyva-González MA, Velázquez-Juárez G, Sanchez-Calderón L, Do Nascimento M, López-Arredondo D, Herrera-Estrella L (2016) A novel genetic engineering platform for the effective management of biological contaminants for the production of microalgae. *Plant Biotechnol J* **14**: 2066–2076.

Michelet L, Lefebvre-Legendre L, Burr SE, Rochaix JD, Goldschmidt-Clermont M (2011) Enhanced chloroplast transgene expression in a nuclear mutant of Chlamydomonas. *Plant Biotechnol J* **9**: 565–574

Park JI, Kent MS, Datta S, Holmes BM, Huang Z, Simmons BA, Sale KL, Sapra R (2011) Enzymatic hydrolysis of cellulose by the cellobiohydrolase domain of CelB from the hyperthermophilic bacterium *Caldicellulosiruptor saccharolyticus. Bioresour Technol* **102**: 5988–5994.

Petersen TN, Brunak S, von Heijne G, Nielsen H (2011) SignalP4.0: discriminating signal peptides from transmembrane regions. *Nat Methods* **8**: 785-786.

Puigbo P, Guzmen E, Romeu A and Garcia-Vallve S (2007) OPTIMIZER: A web server for optimizing the codon usage of DNA sequences. *Nucleic Acids Research* **35**: 126-131.

Sueoka N (1960) Mitotic replication of deoxyribonucleic acid in *Chlamydomonas reinhardtii*. *Proc Natl Acad Sci USA* **46**: 83–91.

Towbin H, Staehelin T, Gordon J (1979) Electrophoretic transfer of proteins from polyacrylamide gels to nitrocellulose sheets: procedure and some applications. *Proc Natl Acad Sci U S A* **76**: 4350-4.

Zheng Y, Zhao J, Xu F, Li Y (2014) Pretreatment of lignocellulosic biomass for enhanced biogas production. *Prog Energy Combust Sci* **42**: 35–53.

Zverlov V, Piotukh K, Dakhova O, Velikodvorskaya G, Borriss R (1996) The multidomain xylanase A of the hyperthermophilic bacterium *Thermotoga neapolitana* is extremely thermoresistant. *Appl Microbiol Biotechnol* **45**: 245–247.
